# Characterisation of Antigen B Protein Species Present in the Hydatid Cyst Fluid of *Echinococcus canadensis* G7 Genotype

**DOI:** 10.1371/journal.pntd.0005250

**Published:** 2017-01-03

**Authors:** Ana Maite Folle, Eduardo S. Kitano, Analía Lima, Magdalena Gil, Marcela Cucher, Gustavo Mourglia-Ettlin, Leo K. Iwai, Mara Rosenzvit, Carlos Batthyány, Ana María Ferreira

**Affiliations:** 1 Cátedra de Inmunología, Facultad de Ciencias/Facultad de Química, Universidad de la República (UdelaR), Montevideo, Uruguay; 2 Laboratório Especial de Toxinologia Aplicada, Center of Toxins, Immune-Response and Cell Signaling (CeTICS), Instituto Butantan São Paulo, Brazil; 3 Unidad de Bioquímica y Proteómica Analíticas, Instituto Pasteur de Montevideo, Montevideo, Uruguay; 4 Instituto de Investigaciones en Microbiología y Parasitología Médica, Facultad de Medicina, Universidad de Buenos Aires, Buenos Aires, Argentina; 5 Departamento de Bioquímica, Facultad de Medicina, Universidad de la República (UdelaR), Montevideo, Uruguay; Istituto Superiore di Sanità, ITALY

## Abstract

The larva of cestodes belonging to the *Echinococcus granulosus* sensu lato (s.l.) complex causes cystic echinococcosis (CE). It is a globally distributed zoonosis with significant economic and public health impact. The most immunogenic and specific *Echinococcus-*genus antigen for human CE diagnosis is antigen B (AgB), an abundant lipoprotein of the hydatid cyst fluid (HF). The AgB protein moiety (apolipoprotein) is encoded by five genes (*AgB1*-*AgB5*), which generate mature 8 kDa proteins (AgB8/1-AgB8/5). These genes seem to be differentially expressed among *Echinococcus* species. Since AgB immunogenicity lies on its protein moiety, differences in AgB expression within *E*. *granulosus* s.l. complex might have diagnostic and epidemiological relevance for discriminating the contribution of distinct species to human CE. Interestingly, *AgB2* was proposed as a pseudogene in *E*. *canadensis*, which is the second most common cause of human CE, but proteomic studies for verifying it have not been performed yet. Herein, we analysed the protein and lipid composition of AgB obtained from fertile HF of swine origin (*E*. *canadensis* G7 genotype). AgB apolipoproteins were identified and quantified using mass spectrometry tools. Results showed that AgB8/1 was the major protein component, representing 71% of total AgB apolipoproteins, followed by AgB8/4 (15.5%), AgB8/3 (13.2%) and AgB8/5 (0.3%). AgB8/2 was not detected. As a methodological control, a parallel analysis detected all AgB apolipoproteins in bovine fertile HF (G1/3/5 genotypes). Overall, *E*. *canadensis* AgB comprised mostly AgB8/1 together with a heterogeneous mixture of lipids, and AgB8/2 was not detected despite using high sensitivity proteomic techniques. This endorses genomic data supporting that *AgB2* behaves as a pseudogene in G7 genotype. Since recombinant AgB8/2 has been found to be diagnostically valuable for human CE, our findings indicate that its use as antigen in immunoassays could contribute to false negative results in areas where *E*. *canadensis* circulates. Furthermore, the presence of anti-AgB8/2 antibodies in serum may represent a useful parameter to rule out *E*. *canadensis* infection when human CE is diagnosed.

## Author Summary

Cystic echinococcosis (CE), a worldwide-spread zoonosis, affects livestock mammals and humans with significant economic and public health impact. It is caused by the infection with the larva of cestodes belonging to *Echinococcus granulosus* complex, a series of parasite species with preference for different hosts. Among them, *Echinococcus canadensis* larva uses mainly camels, goats and pigs as hosts. Species/genotypes belonging to *E*. *canadensis* are considered the second most common cause of human CE, but its contribution may be underestimated since causes asymptomatic or more benign infections than other *E*. *granulosus* complex species. The most relevant antigen for CE diagnosis is a lipoprotein called antigen B (AgB). AgB antigenicity is linked to its protein moiety that is encoded by several genes. One of these genes, *AgB2*, seems to be differentially expressed within *E*. *granulosus* complex. Using high sensitivity proteomic tools we analysed the composition of AgB obtained from *E*. *canadensis* larva, detecting the protein products of all AgB genes, except *AgB2*. Since AgB subunits have been widely used as antigens in immunoassays for human CE diagnosis, our results indicate that using *AgB2* protein product in these assays may lead to false-negative results, particularly in geographical areas where *E*. *canadensis* species/genotypes circulate.

## Introduction

The larval stage (metacestode) of *Echinococcus granulosus* sensu lato (s.l.) causes cystic echinococcosis (CE, traditionally referred to as hydatid disease), one of the most important and widespread parasitic zoonoses. It is a fluid-filled cyst that establishes and grows in the host viscera (mainly liver and lung) of several ungulate livestock (among others sheep, cattle, horse, goat, and pig) and wild animals [1]. Recently, phylogenetic studies have led to split *E*. *granulosus* s.l. into five species, showing preference for infecting different hosts: *E*. *granulosus* sensu stricto (including G1-G3 genotypes), *E*. *equinus* (G4), *E*. *ortleppi* (G5), *E*. *canadensis* (G6–G10) and *E*. *felidis* [2,3]. These species seem to diverge in their transmission dynamics, morphology, rate of development, antigenicity, sensitivity to drugs and, particularly, in their infectivity and pathogenicity in humans, which might therefore influence the design of therapeutic and prophylactic programmes for CE control. This emphasises the need of studies focused on the molecular characterisation and the geographical distribution of *E*. *granulosus* s.l. species/genotypes. *E*. *granulosus* sensu stricto (s.s.) uses mostly sheep as intermediate hosts, but is also capable of infecting other livestock such as cattle as well as humans. Epidemiological studies for examining *E*. *granulosus* s.l. species associated with human CE have determined that *E*. *granulosus* s.s. has an extensive geographical distribution and causes between 73% and 88% of human CE worldwide (reviewed by [4,5]). On the other hand, *E*. *canadensis* G6 and G7 genotypes, which use mainly camels, goats and pigs as intermediate hosts, are also geographically widely distributed and ranked as the second cause of human CE in the world, being responsible for between 11% and 21% of human CE cases according to more recent studies [4–6]. However, these values may be underestimated since *E*. *canadensis* seems to exhibit a lower and/or slower growth than *E*. *granulosus* s.s. in humans, leading to more benign or asymptomatic infections [3,4]. Moreover, in countries such as Austria, Poland, Egypt and Sudan, *E*. *canadensis* is the predominant cause of human CE [3]. Regarding *E*. *canadensis* genotypes, G6 has been preferably associated with human CE but, a recent systematic revision of the species and genotypes of *E*. *granulosus* s.l. responsible for human infections suggests a scenario with a slightly lower prevalence rate for G7 comparing to G6 (9.6% vs 12.2%, respectively) [5]. Interestingly, the geographical distribution of these genotypes differ; G6 genotype is mainly present in human CE cases from America, Asia and Africa whereas the G7 genotype seems to affect mostly some countries in Central Europe. It is worth to mention that there is little or no genotype information on human CE cases reported in many geographical regions/countries, which might influence the epidemiological data cited above.

Despite some progress achieved by prevention campaigns, CE continues being a major public health problem in several countries while represents an emerging or re-emerging disease in others (reviewed by [7,8] and [9–13]). Regarding CE diagnosis, antigen B (AgB), an abundant parasite component present in the HF of the *E*. *granulosus* s.l. metacestode, is the most immunogenic and specific *Echinococcus-*genus antigen. It is a 230 kDa lipoprotein that carries a huge amount of both neutral and polar lipids (around 50% in mass) including fatty acids (FA) and sterols, which *Echinococcus* is not capable of synthesising (reviewed by [14]). This has led to emphasise its hypothetical role in parasite lipid metabolism, taking up host lipids as building blocks for parasite metabolic demands. Moreover, this hypothesis is supported by the fact that AgB belongs to a cestode-specific family of proteins exhibiting ability to bind hydrophobic ligands (HLBP for hydrophobic ligand binding protein) [15,16]. This family has emerged by independent gene expansion events, giving rise to species and gene-specific monophyletic clades. Interestingly, HLBP members are all immunodominant antigens.

AgB antigenicity has been associated with its protein moiety (apolipoprotein components) [17–19] that is encoded by a multigene and polymorphic family with five AgB gene products named *AgB1* to *AgB5* (revised by [20]). The recent assembly of *Echinococcus granulosus* G1 genotype and *E*. *multilocularis* genomes confirmed that this scenario is highly conserved among *Echinococcus* species [21]. The mature protein products of these genes are small (around 8 kDa in mass), α helix-rich secreted polypeptides, with ability to self-assembly generating high-molecular-mass oligomers [22,23]; they are thus named AgB8/1 to AgB8/5 subunits. The native antigen, the recombinant AgB8/1 and AgB8/2 subunits, as well as various synthetic peptides derived from them, have shown to be valuable for CE diagnosis [24–26]; all of them have shown similar diagnostic performance in comparison with crude HF preparations, but in some clinical studies recombinant AgB8 subunits (rAgB8) seem to yield better specificity with little or no loss in sensitivity [27–30].

AgB gene expression in *Echinococcus* s.l. species has been examined suggesting differences between them; this might be relevant for epidemiological investigations intended to discriminate the contribution of distinct *E*. *granulosus* s.l. species to human CE. In the larva of *E*. *granulosus* s.s. all AgB genes were found to be expressed at mRNA level [31], even though only AgB8/1 to AgB8/4 protein products have been certainly detected in HF [23]. On the other hand, no evidence of *AgB5* expression or of the generation of AgB8/2 and AgB8/5 was achieved in *E*. *canadensis* (G6 and G7 genotypes) and *E*. *ortleppi* (G5 genotype) metacestode [32,33]. In particular, *AgB2* was proposed to be a pseudogene in *E*. *canadensis*. In fact, a low-scale sequencing analysis of *E*. *canadensis* genomic DNA, revealed that *AgB2*-related sequences (named EgB2G6v15 to EgB2G6v17 and EgB2G7v15, EgB2G7v18 and EgB2G7v19) contained a substitution at the splicing site (GT-TG instead of GT-AG) that probably interferes with the splicing, leading to the formation of a premature stop codon [32,33]. Taking advantage of the recently available genome of *E*. *canadensis* G7 genotype (published at http://parasite.wormbase.org as echinococcus_canadensis.PRJEB8992.WBPS5.protein), we confirmed the existence of this substitution in ECANG7_10984, which corresponds to the first hit by Blastn analysis using the *E*. *granulosus* s.s. *AgB2* sequence Q27275 as a query at the http://parasite.wormbase.org webpage. However, the generation of a functional AgB2 product may occur by a non-canonical transcriptional mechanism using the TG dinucleotide as splice acceptor site [34]. Studies at transcriptional level failed to identify mRNA coding for a functional AgB2 product in *E*. *canadensis* G7; detected *AgB2* mRNA transcripts were compatible with the use of an upstream AG dinucleotide in the second exon as splice acceptor site that would yield a protein considerably shorter than AgB8/2 due to a premature stop codon [33]. Nevertheless, these studies were carried out using protoscoleces derived from a single cyst of G7 origin (Muzulin et al, 2008), and the germinal layer constitutes a metacestode structure relevant in terms of AgB expression. On the other hand, a deep-sequencing analysis of the transcriptome of *E*. *canadensis* G7 metacestode has not been performed yet. Taken together, the conversion of *AgB2* into a mature and functional product in the larva of *E*. *canadensis* remains uncertain and has not been explored using proteomic tools yet. It is important to remark that predictions based on draft genomes and transcriptional studies are not the ultimate proof of the absence or presence of a protein. Post-transcriptional control of gene expression could play an important role; a gene with low or undetectable expression at the transcriptional level could be efficiently translated allowing the detection of the encoded protein. Various proteomic studies have analysed the parasite and host components present in the HF of *E*. *granulosus* s.l. [23,35,36], nevertheless, none of them provide data about *E*. *canadensis* AgB.

In this work, we have employed high sensitivity proteomic tools to determine the apolipoprotein composition of AgB present in the HF of *E*. *canadensis* G7 genotype. For this proteomic study, we used swine HF as a source of AgB because pigs constitute the main intermediate hosts for *E*. *canadensis* G7 genotype, and HF collects products secreted/excreted by the germinal layer as well as protoscoleces, representing the parasite material where AgB accumulates. Complementary and high-sensitivity approaches including two-dimensional gel electrophoresis (2-DGE) and liquid chromatography (LC) coupled to mass spectrometry (MS) were used as proteomic tools. For a complete biochemical characterisation of *E*. *canadensis* AgB, the lipids carried by the lipoprotein were also examined by high performance thin layer chromatography (HPTLC). Results highlight the concept that AgB is a complex lipoprotein in *E*. *granulosus* s.l. species, including *E*. *canadensis*, being AgB8/1 the predominant apolipoprotein. Furthermore, in contrast with *E*. *granulosus* s.s. [23], AgB8/2 was not detected in *E*. *canadensis* G7 genotype, supporting the concept that *AgB2* is a pseudogene in this species. Since AgB is the most relevant antigen for human CE immunodiagnosis, and the use of rAgB8 subunits offers several advantages for standardising immunoassays (reviewed by [26]), the possible implications of our findings on diagnostic and epidemiological studies on human CE are discussed.

## Materials and Methods

### Parasite material

Fertile hydatid cysts (containing protoscolex, n = 24) were collected from livers of naturally infected pigs during the routine work of local abattoirs in Buenos Aires (Argentina). HF was obtained by aspiration of the content of cysts, and preserved by addition of 5 mM EDTA and 20 μM 3,5-di-tert-butyl-4-hydroxytoluene (BHT) at -20°C until use. Protoscolex were used to analyse parasite genotype on individual cysts. Cyst genotyping was performed by amplification and sequencing of a fragment of the mitochondrial cytochrome c oxidase subunit 1 (COX1) [37]. The sequencing reactions were performed at Macrogen (Korea). All HF samples of swine origin were confirmed to belong to *E*. *canadensis* G7 genotype. For controlling the sensitivity of our proteomics tools, we prepared a pool of bovine HF samples (similarly obtained from local abattoirs in Montevideo, Uruguay). This bovine pool was mainly representative of *E*. *granulosus* s.s. as it contained material from 20 and 3 cysts belonging to *E*. *granulosus* s.s. (18 of G1 and 2 of G3 genotypes) and of *E*. *ortleppi* (G5 genotype), respectively.

### Obtaining AgB from HF

An AgB-enriched fraction was prepared from pooled HF by removing the bulk of host albumin and immunoglobulins by anion exchange chromatography. HF was centrifuged at 10000 x *g* for 20 min at 4°C and the resulting supernatant filtered through 0.45 μm filter membranes (Millipore). The clarified HF (700 mL) was then fractioned by anion exchange chromatography on a Q-Sepharose column (2.5 cm x 10 cm, Pharmacia Biotech, Uppsala, Sweden) previously equilibrated in 20 mM phosphate buffer, pH 7.4 containing 200 mM NaCl, 5 mM EDTA and 20 μM BHT. After washing in equilibration buffer, the retained material was eluted by changing ionic strength to 500 mM NaCl in a single step. The eluted fraction, Q-Sepharose retained fraction (QS_f_), was concentrated 10-times, equilibrated in 20 mM phosphate buffer, pH 7.4 containing 150 mM NaCl, 5 mM EDTA and 20 μM BHT (PBS_EDTA-BHT_), and used to characterise AgB apolipoprotein composition by mass spectrometry as described below. A second purification step was performed based on ultracentrifugation of QS_f_ in a KBr density gradient. Briefly, 2.45 g of KBr were dissolved in 5 ml of QS_f_ in an ultracentrifuge tube and slowly covered with a solution containing 0.15 M NaCl and 0.42 M KBr. After ultracentrifugation (4 h at 332.000 x *g*) two bands were carefully recovered named low (Ld_f_, yellowish-brown band) and high (Hd_f_) density fractions. All fractions were equilibrated in PBS_EDTA-BHT_, and maintained at 4°C under a N_2_ atmosphere until use.

### Identification of AgB8 subunits by 2-DGE plus MALDI-TOF/TOF analysis

2-DGE and MS analysis was performed as described previously [38] but using 150 μg (protein) of the AgB-enriched fraction (QS_f_) for the electrofocusing step in order to detect poorly represented subunits. Briefly, the first dimension was performed with commercially available IPG-strips (7 cm, linear 3–10, GE Healthcare). QS_f_ was prepared and concentrated by using the 2-D Clean-Up kit (GE Healthcare) and dissolved in rehydration solution (7 M urea, 2 M thiourea, 2% CHAPS, 0.5% IPG buffer 3–10 (GE Healthcare), 0.002% bromophenol blue, 17 mM DTT). Samples in rehydration solution were loaded onto IPG-strips by passive rehydration during 16 h at room temperature. The second-dimensional separation (SDS-PAGE) was performed in 15% polyacrylamide gels using a SE 260 mini-vertical gel electrophoresis unit (GE Healthcare). The molecular size marker used was Low Molecular Weight Calibration Kit for SDS Electrophoresis (Amersham GE Healthcare). The gels were colloidal coomassie stained and images were digitalised using a UMAX Power-Look 1120 scanner and LabScan 5.0 software (GE Healthcare). Selected spots were submitted to in gel trypsin digestion (sequencing-grade, Promega) at 37°C overnight. Peptides were extracted from gels using 60% acetonitrile in 0.1% TFA, concentrated by vacuum drying, and then desalted using C18 reverse phase micro-columns (OMIX Pipette tips, Varian). Peptide elution from micro-column was performed directly into the mass spectrometer sample plate with 2 μl of matrix solution (α-cyano-4-hydroxycinnamic acid in 60% aqueous acetonitrile containing 0.1% TFA). Mass spectra of digestion mixtures were acquired using a matrix-assisted laser desorption/ionization time-of-flight mass spectrometer (MALDI-TOF/TOF, 4800 Analyzer, ABi Sciex) in positive reflector mode and were externally calibrated using a mixture of peptide standards (Mix 1, ABi Sciex). Collision induced dissociation (CID) MS/MS spectra of selected peptides ions were also acquired. Proteins were identified with measured m/z values in MS and MS/MS acquisition modes and using the MASCOT search engine (Matrix Science, http://www.matrixscience.com) in the Sequence Query search mode. AgB8 subunits were identified by searching in both, the NCBInr and an in-house *Echinococcus* databases using the following search parameters: unrestricted taxonomy, monoisotopic mass tolerance, 0.05 Da; fragment mass tolerance, 0.2 Da; carbamidomethyl cysteine and methionine oxidation as variable modifications and up to one missed tryptic cleavage allowed. Significant protein scores (*p* < 0.05) were used as criteria for positive protein identification. In addition, at least two unique peptides with ion significant score (*p* < 0.05) were required for AgB8 subunit identification. The in-house *Echinococcus* database was built comprising all sequences of *E*. *canadensis* (G7 genotype, published in http://parasite.wormbase.org as echinococcus_canadensis.PRJEB8992.WBPS5.protein) and of *E*. *granulosus* s.s. (G1 genotype, published in www.genedb.org as EGU_proteins_29042013_products.fa) plus a total of 102 full length sequences, including polymorphic variations at the level of the AgB mature products as well as the orthologous products in other *Echinococcus* species (available on NCBInr, March 2015). Furthermore, to study *E*. *canadensis* AgB8/2 presence, we took into account the previous characterisation of this gene (at DNA and mRNA level, [32]) and added to the database those protein sequences that would be generated by non-canonical splicing of *E*. *canadensis AgB2*-related sequences EgB2G6v15 to EgB2G6v17, EgB2G7v15, EgB2G7v18 and EgB2G7v19, as well as of *AgB ECANG7_10984* gene (in all putative open reading frames, S1 Appendix).

### Identification of AgB8 subunits by LC-MS/MS analysis

Samples (QS_f_) were analysed by LC tandem-mass spectrometry (LC-MS/MS) using five analytical replicates. Proteins were reduced, carbamidomethylated, and digested in solution with sequencing-grade trypsin (Sequencing-grade Promega; 1:50 enzyme to total protein ratio) in 70 mM ammonium bicarbonate pH 8.0 buffer containing 2 M guanidine hydrochloride for 12 h at 37°C. Peptides were further concentrated, desalted using C18 reverse phase micro-columns (OMIX Pipette tips, Varian) and eluted with 60% aqueous acetonitrile containing 0.1% TFA. Peptide mixtures were dried and resuspended in 5% aqueous acetonitrile containing 0.1% formic acid. Five micrograms of each sample were analysed in an EASY-nLC II nanoflow liquid chromatography (Thermo Fisher Scientific, USA) coupled to a LTQ-Orbitrap Velos mass spectrometer (Thermo Fisher Scientific). Peptide mixture was injected into a trap column (I.D. 100 μm x O.D. 360 μm x 50 mm) packed with Jupiter C18 10 μm beads (Phenomenex Inc., USA) for desalting with 100% solvent A (0.1% formic acid). Peptides were then fractionated on an analytical column (I.D. 75 μm x O.D. 360 μm x 100 mm) packed in-house with Aqua C-18 5 μm beads (Phenomenex Inc.) at a flow rate of 200 nL/min using a 60 min linear gradient from 5 to 35% of solvent B (0.1% formic acid in acetonitrile). Afterwards, a gradient from 35 to 85% of B in 5 min was applied for ensuring a complete elution. Nano-electrospray voltage was set to 2.3 kV, the source temperature to 250°C and mass spectrometer was operated in a data-dependent acquisition mode, where the top ten precursor ions in each cycle were selected for fragmentation event by CID. Ion trap injection time was set to 100 ms and FT-MS injection time was set to 1000 ms with a resolution of 60,000 across m/z 300–1800. For IT scans, fragmentation was carried out on ions above a threshold of 200 counts, and dynamic exclusion was enable with an exclusion list size of 500 for 90 seconds, repeat duration of 30 seconds and a repeat count of 1. Raw mass data files (.raw) were analysed in Maxquant (v.1.5.5.1) and its built-in Andromeda search engine. Parasite and host proteins were identified using MaxQuant software by searching MS and MS/MS data against a merged database comprising the *Echinococcus* database (built as described above) and the *Bos taurus/Sus scrofa* database (downloaded from UniProt, April/2016). Trypsin was set for enzyme specificity with a maximum of two missed cleavages, mass tolerance for precursor ions was set to 10ppm, and fragment ion mass tolerance was set to 0.5 Da. MS/MS spectra searches incorporated fixed modifications of carbamidomethylation of cysteine, oxidation of methionine and protein N-terminal acetylation were set for variable modifications. Maximum false peptide and protein discovery rate was set to 0.01. Proteins matching to the reverse database were eliminated. Statistical analysis for protein identification was performed using Perseus (v. 1.4.0.11) based on unique peptides MS intensities, the presence of a minimum of two unique peptides and PEP (posterior error probability) < 0.01. To evaluate the abundance of each AgB protein species (AgB8 subunit) the intensity-based absolute quantification (iBAQ) was used as it has been reported as a useful label-free quantification method provided by MaxQuant. In the iBAQ algorithm the sum of all identified peptide intensities (maximum detector peak intensities of the peptide elution profile, including all peaks in the isotope cluster) is divided by the number of theoretically observable tryptic peptides, and expressed as log_2_ values [39]; this operation transforms a measure that is expected to be proportional to mass (intensity) into one that is proportional to molar amount (iBAQ). To determine the relative abundance of each AgB8 subunit in AgB (riBAQ_AgB_), we divided the iBAQ value corresponding to each AgB8 subunit by the sum of the iBAQ values obtained for all AgB8 subunits, and expressed this ratio as a percentage. Replicate results were merged with Perseus and values for iBAQ, riBAQ_AgB_, score and the percentage of the protein sequences covered by identified peptides (% CO) are expressed as the mean of all runs (n = 5). The total number of the identified peptide spectra matched for a protein (PSM) was also estimated as the sum of all runs. Only proteins present in at least 3 of the 5 analytical replicates were considered as positively identified.

### Lipid extraction and analysis

AgB total lipids were analysed using Ld_f_ (between 0.25 and 0.5 mg of protein) following the methodology that we have already described [38]. Qualitative analysis of lipid classes was performed by HPTLC using double development for neutral and polar lipids as described previously [38], but lipid bands were visualised under iodine vapour. Identification of lipid classes was performed by comparison with primary and secondary standards run on the same HPTLC plate.

## Results

### Apolipoprotein composition of AgB present in *E*. *canadensis* HF

The apolipoprotein composition of AgB present in fertile HF of *E*. *canadensis* G7 genotype was analysed using MS based methodologies. For this purpose, we prepared a biological representative pool of *E*. *canadensis* HF from 24 individual swine cysts, each one of G7 origin according to COX1 genotyping. However, to achieve an adequate sample for AgB apolipoprotein characterisation, we firstly carried out an enrichment step since AgB is poorly represented in HF compared to host albumin and immunoglobulins. Taking advantage that AgB can be selectively separated from these host proteins employing a Mono-Q [40] or Q-Sepharose beads [41], we prepared an AgB enriched-fraction by a single step anion exchange chromatography of HF on Q-Sepharose; this step concentrates AgB favouring the detection of lower represented apolipoproteins. S1 Fig shows the SDS-PAGE analysis of fractions obtained by this chromatography. As expected, AgB was retained by Q-Sepharose beads and eluted with 500 mM NaCl pH = 7.4 (fraction QS_f_) since QS_f_, but not the flow through fraction (FT_f_), showed 8, 16 and 24 kDa bands in agreement with the typical AgB ladder-like pattern (S1 Fig, small head arrows) [42]. In contrast, the majority of the most abundant host proteins present in HF (albumin and immunoglobulins) did not bind to Q-Sepharose, being recovered in FT_f_ (S1 Fig). Additional steps based on ultracentrifugation on a KBr density gradient achieved to purify AgB (see below). Nevertheless, since these purification steps led to protein losses, we rather to use QS_f_ for characterising AgB protein species; we cannot ruled out that AgB includes particles of different densities and/or less abundant AgB apolipoproteins would be not represented in the purified AgB preparation (Ld_f,_ see below).

**Fig 1:**
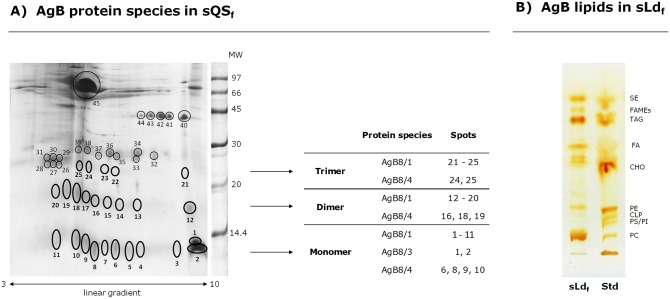
Composition of native AgB: sQS_f_ apolipoproteins identification by 2-DGE plus MALDI-TOF/TOF and sLd_f_ lipid moiety analysis. **A)** Analysis of sQS_f_ by 2-DGE (Figure is representative of analytical triplicates), using a 3–10 lineal gradient of pH in the first dimension, and a 15% polyacrylamide gel for SDS-PAGE in the second dimension. Gels were stained with colloidal coomassie. The presence of host and parasite components was studied by analysing all spots by MS (MALDI-TOF/TOF). AgB was found in spots regularly spaced at around 8, 16 and 24 kDa (bold circles and arrows). The table illustrates which AgB8 subunits were identified in spots corresponding to the monomeric, dimeric and trimeric forms of AgB. MW: molecular weight (KDa). **B)** Analysis of sLd_f_ by HPTLC (Figure is representative of analytical triplicates). Standards and samples (about 10 μg) were applied onto HPTLC plates and resolving using double development solvent system for characterisation of both neutral and polar lipid classes. Lipid bands were visualised using iodine vapour. Std: standard containing polar and neutral lipids; PC: phosphatidylcholine; PS: phosphatidylserine; PI: phosphatidylinositol; CLP: cardiolipin and PE: phsophatidylethanolamine; CHO: cholesterol; FA: free fatty acids; TAG: triacylglycerols; FAMEs: fatty acid methyl esters; SE: sterol esters.

The presence of AgB8 subunits in swine QS_f_ (sQS_f_) was examined by 2-DGE followed by MALDI-TOF/TOF MS. As shown in Fig 1A, AgB was detected in several spots corresponding to the monomer, dimer and trimer (indicated with bold circles and numbers in the Fig). Interestingly, the monomeric as well as oligomeric AgB forms comprised several components spread over a wide range of pH (between 9.4 and 4.5). AgB protein species identified in those spots included AgB8/1, AgB8/3 and AgB8/4 subunits (Fig 1A and S1 Table). AgB8/1 was the predominant subunit detected in all spots belonging to AgB (n = 25); the presence of both Q86BY8 and Q3YFQ5 isoforms is plausible accordingly to the set of unique peptides identified by MALDI-TOF/TOF (Table 1). Q86BY8 and/or Q3YFQ5 are basic proteins (pI = 9.11) that would agree with their identification in spots focused at around pH 9.4 (named #1 and #2, Fig 1A), but not in more acidic ones. Similarly, two AgB8/4 protein species with a theoretical pI of 6.15 (named Q6J0W7 and Q6Q0G2, Table 1) were detected in 9 spots focused in a wide range of pH (Fig 1A and S1 Table). Thus, these results suggest the presence of post-translational modifications in both AgB8/1 and AgB8/4. Neither signals corresponding to phosphorylated peptides nor the formation of carbonyl groups in AgB8 subunits (because of oxidative reactions with oxides of nitrogen or metal catalysed oxidation) were detected by MS and Western Blot, respectively (S2 Appendix). Thus, further studies are needed to elucidate which molecular modifications explain the AgB pattern obtained by 2-DGE. On the other hand, AgB8/3 was identified in spots #1 and #2 based on various unique peptides (Table 1 and S1 Table); while these peptides cannot distinguish between the AgB8/3 isoforms named Q6VXZ8 and Q6VXZ9, the presence of Q6VXZ8 seems to be more likely according to its pI. Finally, AgB8/2 and AgB8/5 were not detected in sQS_f_.

**Table 1:** AgB8 protein species identified in sQS_f_ and bQS_f_ by 2-DGE followed by MALDI-TOF/TOF.

	Uniprot Accession Number	MW (Da) / pI	sQS_f_	bQS_f_	Unique peptides in some polymorphic isoforms^#^	Unique peptides shared by all isoforms
**AgB8/1**	**Q86BY8**^a^ **Q3YFQ5**	**7492.8 / 9.1** **7518.8 / 9.1**	+++	**-**	***ELVAEGK***	DDGLTSTSR, YFFER, YFFERDPLGQK, DPLGQK, VVDLLK, ELEEVFQLLR
	Q5EKQ4 Q9UA06^b^	7589.9 / 8.3 7555.9 / 8.3	-	+++	*MFGEVK*	
**AgB8/2**	Q5EKP1 Q27275^c^	7906.2 / 9.4 8193.5 / 9.4	-	+	-	MGQVVKK, RWGELR, DFFRNDPLGQR, NDPLGQR
**AgB8/3**	**Q6VXZ8**^d^ **Q6VXZ9**	**7708.0 / 8.8** **7709.0 / 6.8**	+	**-**	***HFFQSDPLGR*, *ELASVCQVVR ELASVCQVVRK***	
	Q95NW6^e^ A0A068X006-1	7858.2 / 8.0 6712.8 / 6.8	-	n.c.d	*HFFQSDPLGKK*, *DVACVCEMVR*	
**AgB8/4**	**Q6J0W7**^f^ **Q6Q0G2**	**8353.7 / 6.2** **8337.6 / 6.2**	++	**-**	***CLITR*, *KLSEVR*, *SDPLGQR YVKDLLEEEEEEDDSK, DLLEEEEEEDDSK***	DLTAICQK
	Q6UZE2^g^ Q6UZD8 Q6UZE3^h^	8199.6 / 6.8 8252.7 / 8.0 8171.6 / 6.8	-	++	*KLGEIR*, *VHEVLKK, YVKDLLEEEDEDDLK DLLEEEDEDDLK*	

Superscript letters indicate alternative names for the protein product as follows:

^a^ Q3YFQ4;

^b^ U6JQF4 and Q5S577;

^c^ Q5EKN4, C1KBK4, Q6Q0H3 and Q6Q0I3;

^d^ Q3YFP8;

^e^ Q5EKQ8, Q5EKR3 and Q95W92;

^f^ Q6Q0G7;

^g^ Q6GYC5;

^h^ D1MH02

Molecular mass (MW) and isoelectric point (pI) of mature proteins were calculated using the "compute pI/MW" Expasy tool (http://web.expasy.org/compute_pi/)

^+^ proteins found in one or two spots;

^++^ proteins found in 3 to 12 spots;

^+++^ proteins identified in more than 12 spots

n.c.d. means not confirmed detection. Signals corresponding to unique peptides were found, but the identity of these peptides could not be confirmed by fragmentation in MALDI MS/MS analysis

^#^ peptides that allowed differentiating polymorphic isoforms within a particular AgB subunit are indicated in *italic* letter

The presence of AgB8/2, but not AgB8/5, has been previously reported in bovine HF collected from *E*. *granulosus* s.s. cysts [23]. We performed thus a similar study by 2-DGE plus MALDI-TOF/TOF using a pool of bovine HF (mostly belonging to *E*. *granulosus* s.s. according to COX1 genotyping) to evaluate whether our proteomic tool reached enough sensitivity for AgB8/2 analysis. Results showed the presence of AgB8/2, but not of AgB8/5 in bovine QS_f_ (bQS_f_, Table 1, S3 Appendix) in accordance with the earlier report. Overall, detection of AgB8/2 in bQS_f_ but not in sQS_f_ suggests that AgB8/2 is not present in G7-HF.

In addition to AgB protein species, analysis of sQS_f_ and bQS_f_ by 2-DGE plus MALDI-TOF/TOF showed the presence of *Echinococcus* Ag5 (22 and 38 KDa subunits) as well as of some host components (remaining albumin and immunoglobulin light chains, as well as apolipoprotein A-I (Apo A-I); see S1 Table and S3 Appendix).

Confirmation of the observations described above was achieved using LC-MS/MS since this methodology enables a high sensitivity quantitation of proteins in complex biological samples. Results are summarised in Table 2, in which the iBAQ parameter (expressed as log_2_ values) is proportional to the protein molar amounts while the riBAQ_AgB_ refers to the relative abundance of each protein species in AgB. Analysis of sQS_f_ showed that AgB8/1 was the major protein component, representing 71% of total AgB apolipoproteins followed by AgB8/4 (15.5%), AgB8/3 (13.2%) and AgB8/5 (0.3%). AgB8/2 was not detected in sQS_f_ although our database included all AgB8/2 sequences available for *E*. *granulosus* s.l. species (comprising those protein products that could be generated because of non-canonical splicing of all available *AgB2* related sequences). As expected, *E*. *granulosus s*.*s*. AgB8/2 was identified in bQS_f_. Because the bovine HF pool contained samples from *E*. *ortleppi* G5 genotype, we looked for *E*. *ortleppi* AgB8/2 specific peptides in bQS_f_ with no success. This may be consequence of the low proportion of *E*. *ortleppi* components in the bovine HF pool (around 15% of the total volume), or of the lack of *AgB2* functionality in this species, as proposed previously [32]. On the other hand, we detected in sQS_f_ two unique peptides that make reliable the identification of AgB8/5 in *E*. *canadensis* metacestode (Table 2), contrasting with previous findings at the RNA level [33]. This contrast can be explained by the fact that *AgB5* would be poorly expressed in the metacestode and/or that previous mRNA expression studies were performed using primers, which were not specifically designed for *E*. *canadensis AgB5*. On the other hand, our results endorse previous data at mRNA [31] and protein levels [35] for *AgB5* expression in *E*. *granulosus* s.l. metacestode. Finally, the detection in sQS_f_ of AgB8/5, an AgB subunit barely expressed in the metacestode of *Echinococcus* species, denotes the high sensitivity reached in our proteomic study.

**Table 2:** AgB8 protein species identified in sQS_f_ and bQS_f_ by LC-MS/MS.

	Uniprot Accession Number	MW (Da) /pI	Swine QS_f_ (G7 genotype)				Bovine QS_f_ (G1, G3, G5 genotypes)				AgB unique peptides		
			iBAQ	riBAQ_AgB_	Score	%CO	iBAQ	riBAQ_AgB_	Score	%CO	only detected in isoforms of a particular species	shared by isoforms of different species	
**AgB8/1**	**Q86BY8**^a^ **Q3YFQ5**	**7492.8 / 9.1** **7518.8 / 9.1**	**27.7**	**71.0**	**131.7**	**55.0**	**-**	**-**	**-**	**-**	*nf*	DPLGQKVVDLLKELEEVFQLLR, ELEEVFQLLR, ELEEVFQLLRK, VVDLLKELEEVFQLLR, VVDLLKELEEVFQLLRK	DDGLTSTSR, YFFERDPLGQK, DPLGQKVVDLLK
	Q5EKQ4 Q9UA06^b^	7589.9 / 8.3 7555.9 / 8.3	-	-	-	-	29.1	92.2	162.3	72.6	SVMKMFGEVK, VVDLLKELEEVFQLLRKMFGE, VKYFFER, GLIAEGE		
	Q3YFP9	7476.7 / 9.1									*nf*	*nf*	
**AgB8/2**	Q5EKP1 Q27275^c^	7906.2 / 9.4 8193.5 / 9.4	-	-	-	-	20.9	0.3	75.8	60.6	AHMGQVVK, AHMGQVVKK, DFFRNDPLGQR, NDPLGQR, LVALGNDLTAICQK, YVKNLVEEK, YVKNLVEEKDDDSK, NLVEEKDDDSK	*nf*	*nf*
**AgB8/3**	**Q6VXZ8**^d^ **Q6VXZ9**	**7708.0 / 6.8** **7709.0 / 6.8**	**25.2**	**13.2**	**53.6**	**49.2**	-	-	-	-	ELASVCQVVR	DDDDEVTK, HFFQSDPLGR, NLLDEAE	*nf*
	Q3YFP3	7740.1 / 8.8	-	**-**	**-**	-	22.5	1	36.1	24.6	ELASVCQMVR		*nf*
	Q95NW6^e^ A0A068X006-1	7858.2 / 8.0 6712.8 / 6.8	-	**-**	**-**	-	25.2	6.3	49.6	50.2	DDDDDEVTK, DVASVCEMVR, HFFQSDPLGK, HFFQSDPLGKK	*nf*	*nf*
**AgB8/4**	**Q6Q0G7**^f^ **Q6Q0G2**	**8353.7 / 6.2**	**25.5**	**15.5**	**55.0**	**42.2**	**-**	**-**	**-**	**-**	DFFRSDPLGQR, YVKDLLEEEEEEDDSK, DLLEEEEEEDDSK	*nf*	DLTAICQK
	Q6UZE2^g^ Q6UZD8	8199.6/ 6.8 8252.7 / 8.0	-	**-**	**-**	-	18.4	0.05	58.6	78.6	SDPLGQKLVALGR	LGEIRDFFR, DFFRSDPLGQK, SDPLGQK, LQLKVHEVLK, VHEVLKK, YVKDLLEEEDEDDLK, DLLEEEDEDDLK	
	Q6UZE3^h^	8171.6 / 6.8	-	**-**	**-**	-	19.0	0.08	77.0	76.8	SDPLGQKLAALGR		
**AgB8/5**	**Q1EQ64**	**7455.7 / 8.1**	**19.8**	**0.3**	**12.2**	**8.0**	-	-	**-**	**-**	*nf*	*nf*	DFFLLAR EFFASDPMGQK
	D1MH21^i^	7499.7 / 8.1	-	**-**	**-**	-	18.7	0.07	13.3	17.2	*nf*	*nf*	

Accordingly to *E*. *canadensis* genome (available at http://parasite.wormbase.org), protein species identified as Q86BY8/Q3YFQ5, Q6Q0G7/Q6Q0G2 and Q1EQ64 are products of ECANG7_10738, ECANG7_09982 and ECANG7_10674 genes, respectively

The superscript letters indicate that the protein has also been annotated as follows:

^a^ Q3YFQ4;

^b^ U6JQF4 and Q5S577;

^c^ Q5EKN4, C1KBK4, Q6Q0H3 and Q6Q0I3;

^d^ Q3YFP8;

^e^ Q5EKQ8, Q5EKR1, Q5EKR3 and Q95W92;

^f^ Q6J0W7;

^g^ Q6GYC5;

^h^ D1MH02;

^i^ U6JQF8 and Q1EQ65

Values in bold correspond to swine-origin material (sQsf)

Molecular mass (MW) and isoelectric point (pI) of mature proteins were calculated using the "compute pI/MW" Expasy tool (http://web.expasy.org/compute_pi/)

iBAQ: values were log2-transformed and are given as median of quintuplicates with a relative standard deviation ≤ 1.1%

riBAQAgB: Relative abundance of each protein species in AgB

% CO: Coverage values, percentage of the protein sequence that is covered by the identified peptides

n.f: not found

### Lipid classes present in AgB purified from HFs

AgB is likely involved in taking up host lipids, which are essential for *Echinococcus* spp., as building blocks for parasite needs [14]. For a complete biochemical characterisation, we purified *E*. *canadensis* AgB from QS_f_ and characterised the lipid classes present in its lipid moiety. AgB purification was performed by a novel procedure based on density-gradient ultracentrifugation; this method preserves AgB native structure and yields AgB particles independently of its apolipoprotein composition. AgB was mainly recovered in the low density fraction, Ld_f_, but consecutive ultracentrifugation rounds were needed to achieve a good-quality AgB preparation (about 95% pure, according to SDS-PAGE, S1B Fig), although these steps goes against the final AgB yield. Several lipid classes including highly polar (phosphatidylcholine and, to a lesser extent, phosphatidylethanolamine) and neutral lipids (sterols, free FA, triacylglycerols and sterol esters) were detected in *E*. *canadensis* AgB (Fig 1B), just as we have already described for AgB immunopurified from bovine pooled HF ([38] and S3 Appendix). In sum, the observed differences in the protein composition of AgB preparations from distinct *E*. *granulosus* s.l. species (Table 2), did not affect the lipid class composition of the lipoprotein. However, qualitative and or quantitative differences in lipid components within each class cannot be excluded and require further studies.

### *Echinococcus* and host proteins related to lipid metabolism in HF

The proteomic analysis of sQS_f_ and bQS_f_ by LC-MS/MS allowed identifying several parasite and host proteins in HF (S4 Appendix). Regarding parasite proteins, we identified several proteins with putative diverse functions, but taking into account the iBAQ values in both samples, AgB subunits were found to be the most abundant components, followed by Ag5. In particular, we significantly identified in sQS_f_ and/or bQS_f_ parasite proteins with a potential role in lipid metabolism. One of the most interesting was an *E*. *granulosus* s.s. HLBP detected in bQS_f_ (*Eg*HLBP, Uniprot protein accession A0A068WMS7_EGHR). The gene that encodes *Eg*HLBP, referred to as EgrG_000549200 (http://www.genedb.org/), mapped outside AgB cluster [43]. This novel *Eg*HLBP has a higher similarity to an uncharacterised protein of *E*. *granulosus* s.s. (Uniprot protein accession W6UNU2_ECHGR, 87% identity) and to *Taenia solium* HLBP1 and HLBP2 (Uniprot protein accession G3FJ94_TAESO and G3FJ95_TAESO, with 68% and 71% of identity, respectively) than to AgB (45% identity); the alignments of *Eg*HLBP with these proteins are shown in S5 Appendix. Interestingly, we detected *Eg*HLBP in HF, while both *Ts*HLBP1 and *Ts*HLBP2 were not detected by Western Blot in the metacestode and showed a high expression in the *T*. *solium* adult [44]. In addition, we identified the Lipid transport protein N-terminal in both sQS_f_ and bQS_f_. This protein exhibits only significant similarity with its orthologous in *E*. *multilocularis* (96% identity) and with an *E*. *granulosus* s.s. apolipophorin (Uniprot accession W6UHB7_ECHGR, 98% identity), neither of which have been characterised yet. All of them are high MW macromolecules, containing various conserved regions found in several lipid transport proteins, including vitellogenin, microsomal triglyceride transfer protein and apolipoprotein B-100 (Smart accession number SM00638; PROSITE PS51211). Finally, we identified various host apolipoproteins, including Apo A-I, in sQS_f_ and bQS_f_. Apo A-I has previously been detected in *E*. *granulosus* s.l. HF [35]. Interestingly, Apo A-I and an Apo A-I binding protein (EmABP) were found to be present in HF of *E*. *multilocularis* [45]. In our study, EmABP orthologues in *E*. *granulosus* s.l. species were not found in QS_f_ or bQS_f_, but their presence in HF requires further investigation.

## Discussion

This work contributes to widen the information available on *E*. *granulosus* s.l. AgB subfamilies, particularly at the level of the presence and abundance of their protein products in the metacestode of *E*. *canadensis* G7 genotype. Using high sensitivity and quantitative proteomic analysis of a representative number of hydatid cysts, we showed that AgB8/1 is the major AgB apolipoprotein in the HF of *E*. *canadensis* G7 genotype. This strengthens the concept of AgB8/1 predominance in the HF of various *E*. *granulosus* s.l. species [23,35]. Since AgB likely contributes to the mechanisms used by the metacestode to transport lipids, particularly those that the parasite is unable to synthesise, this result would indicate that AgB8/1 is the main AgB apolipoprotein involved in this transport, and, in consequence, the presence of AgB8/1 receptors in parasite and host cells is worth to be further studied. To this respect, AgB8/1 was found to bind selectively to monocyte and macrophages, but the molecular partners involved have not been identified yet [41]. Furthermore, we identified an additional *Echinococcus* HLBP (EgrG_000549200) and host apolipoproteins (particularly Apo A-I) in QS_f_, which suggests that several lipid carriers are involved in parasite mechanisms aimed at providing essential lipids to metacestode tissues. However, taken into account their abundance in HF (iBAQ values), their contribution to lipid transport within metacestode tissues seems to be lower than that of AgB.

On the other hand, our results support a differential expression of *AgB2* among *E*. *granulosus* s.l. species; AgB8/2 was not detected in the HF of *E*. *canadensis* G7 genotype contrasting with their detection in *E*. *granulosus* G1 genotype ([23] and this work). Despite this difference, we did not found significant differences in the lipid moiety of AgB purified from sQS_f_ and bQS_f_, at least in terms of lipid classes. This may be a result of the fact that AgB8/2 showed a low relative abundance in comparison with AgB8/1 in *E*. *granulosus* s.s., and that no differences have been observed between the lipid binding properties of AgB8/1 and AgB8/2 using *in vitro* assays [46]. Regarding to the lack of AgB8/2 in *E*. *canadensis*, it would be explained at the molecular level by the occurrence of an A/T transversion at the splicing site that likely interferes with canonical splicing mechanisms and the synthesis of a functional protein product. Bearing in mind that AgB is diagnostically valuable for human CE, differences in AgB apolipoprotein composition between *E*. *granulosus* s.s. and *E*. *canadensis* are informative for diagnosis and epidemiological studies on this zoonosis. In particular, rAgB8/1 and rAgB8/2 subunits, as well as peptides derived from them, have yielded reasonable diagnostic performance in immunoassays using panels of sera from patients with CE and other helminth infections (reviewed by [26]). In most of these studies rAgB subunits were assessed as single antigens, although combination between them (AgB subunit cocktail) or with other HF antigens would help to achieve more sensitive and specific tests [27,47–51]. In any case, our results indicate that the use of AgB8/2 as antigen in immunoassays might contribute to false-negative results in patients infected by *E*. *canadensis* G7 genotype. Probably the same holds for infections with *E*. *canadensis* G6 genotype, although the lack of AgB8/2 at the protein level requires confirmation. As we have mentioned above *E*. *canadensis* accounts for between 11% and 21% of CE human cases worldwide reaching a higher prevalence in some countries, therefore, our results denote the importance of adapting the diagnostic tools to the epidemiological situation of each geographical region. Taking into account the global distribution of *E*. *canadensis*, our observations may thus be of major importance for regions where cases of human infection by *E*. *canadensis* have been reported (including countries in all continents, such as Argentina, Egypt, Iran, Kenya, Mauritania, Mongolia, Poland, South Africa and ex-Yugoeslavia, [4,5]). However, because of the scarce information about the *E*. *granulosus* species/genotypes associated with human CE in many countries/regions, our data might be of interest for any region where *E*. *canadensis* is known to circulate (for instance, Southern Brazil and the Mediterranean region [52,53]).

The determination of *E*. *granulosus* s.l. genotype/species responsible for human CE cases is a subject of relevance since, as we have already mentioned, species belonging to *E*. *granulosus* complex differ in biological features (*i*.*e*. infectivity and pathogenicity in humans), influencing control program design as well as disease follow-up and treatment [54]. Genotyping is thus a critical task. However, nowadays, it can only be performed after surgery in order to obtain parasite samples, and using molecular biology tools. Since *E*. *granulosus* s.s. and *E*. *canadensis* are the most common cause of human CE, it would be worth to develop simple tools to differentiate the infections caused by them. The presence of antibodies against AgB8/2 in serum would be easy to determine through conventional immunoassays based on the use of rAgB8/2 or AgB8/2-derived peptides as antigen. Thus, including this kind of immunoassays during routine diagnosis of human CE would allow ruling out *E*. *canadensis* infection based on the presence of anti-AgB8/2 antibodies.

## Supporting Information

S1 Fig AgB purification from HF. **A)** Analysis by SDS-PAGE of the fractions obtained by anion exchange chromatography on Q-Sepharose of HF and subsequent ultracentrifugation in a KBr gradient (Figure is representative of analytical triplicates). Between 25 and 30 μg of each fraction were separated on a 15% polyacrylamide gel in reducing conditions (6 mM DTT). Notice that the bulk of host albumin (band of apparent MW 65 kDa, indicated with a big head arrow) and immunoglobulins (bands with apparent MW of about 50 and 25 kDa corresponding to the high and light chains of IgG, respectively, indicated with bold arrows) present in HF were not retained on the Q-Sepharose column and appeared in the flow through fraction (FT_f_). In addition, an around 8 kDa band (containing likely AgB8 subunits) was slightly stained in HF and undetectable in FT_f_, but became prominent in the fraction retained by the Q-Sepharose column (QS_f_). Moreover, the typical AgB pattern with regularly spaced bands is observed in QS_f_ (small head arrows). After the first ultracentrifugation round of QS_f_, the high (Hd_f_) and low (Ld_f_) density fractions were recovered. Ld_f_ contains mainly AgB. **B)** Analysis of SDS-PAGE of Ld_f_ obtained by two consecutive ultracentrifugation rounds (indicated as 1^st^ UC and 2^nd^ UC) showing that at least two rounds were needed to achieve a good-quality AgB preparation. Simple arrows indicate AgB monomeric and oligomeric forms.
(TIF)

S1 Table (PDF)

S1 Appendix Putative protein products of *E*. *canadensis* AgB2. Nucleotide sequence for AgB2 (ECANG7_10984) were obtained from *E*. *canadensis* genome available at http://parasite.wormbase.org. Putative products of ECANG7_10984 were predicted using the Expasy translate tool (http://web.expasy.org/cgi-bin/translate/dna_aa).
(PDF)

S2 Appendix Analysis of post-translational modifications: phosphorylation and formation of carbonyl groups in AgB subunits. (PDF)

S3 Appendix Characterisation of bovine AgB. bQS_f_ proteins species were analysed by DGE followed by MALDI-TOF/TOF while the lipid moiety of bLd_f_ was analysed by HPTLC.
(PDF)

S4 Appendix *Echinococcus* and host proteins identified in sQS_f_ and bQS_f_ by LC-MS/MS. (PDF)

S5 Appendix Alignments of *Echinococcus* HLBPs named A0A068WMS7_EGHR and W6UNU2_ECHGR with their orthologous in *Taenia solium* and *E*. *granulosus* AgB subunits. (PDF)
