# Supplementary figures and images for: Characterisation of Antigen B Protein Species Present in the Hydatid Cyst Fluid of Echinococcus canadensis G7 Genotype
Source: PLoS Negl Trop Dis. 2017 Jan 3;11(1):e0005250. doi: 10.1371/journal.pntd.0005250 (PMC5234841; doi:10.1371/journal.pntd.0005250)

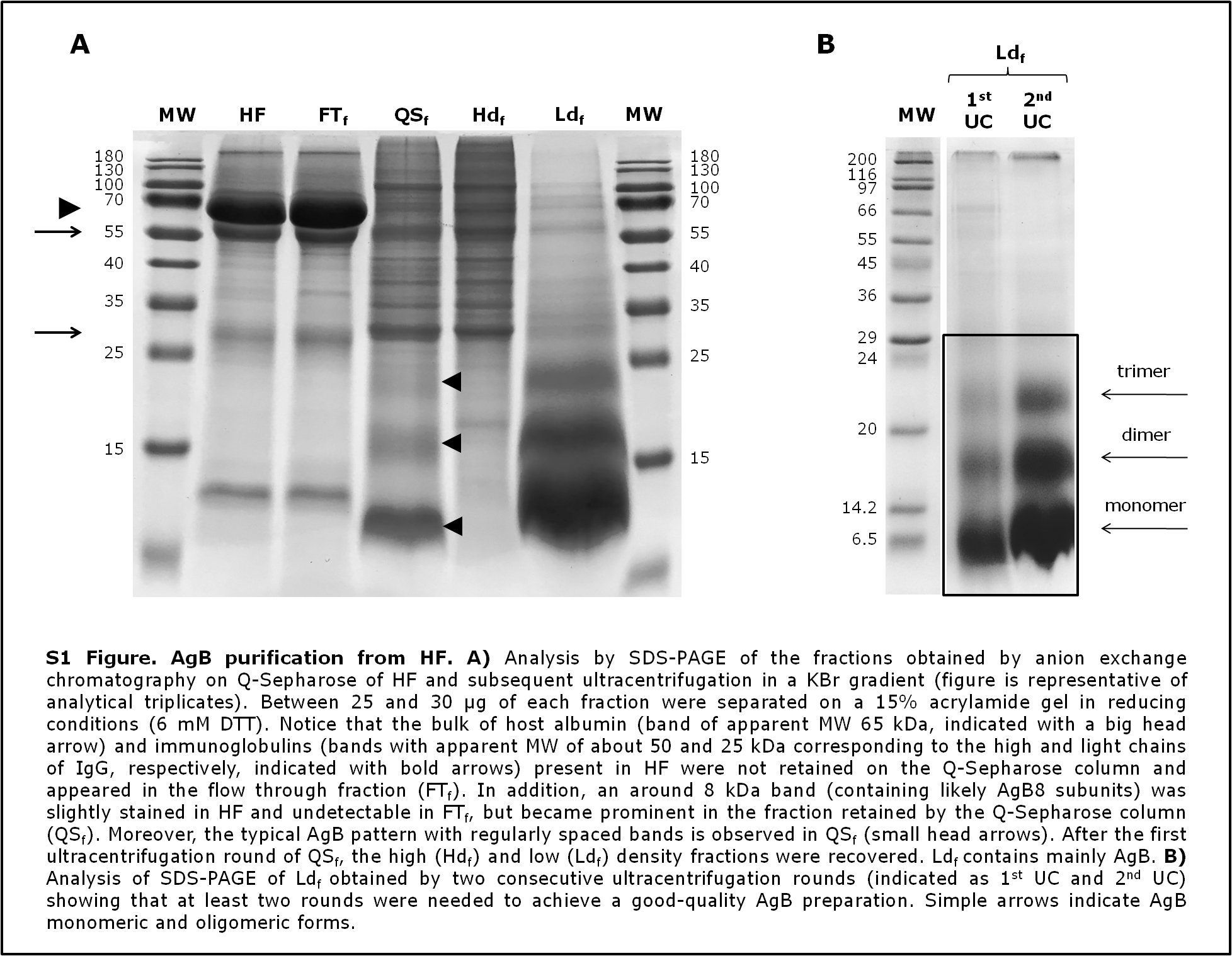

Supplement: S1 Fig — A) Analysis by SDS-PAGE of the fractions obtained by anion exchange chromatography on Q-Sepharose of HF and subsequent ultracentrifugation in a KBr gradient (Figure is representative of analytical triplicates). Between 25 and 30 μg of each fraction were separated on a 15% polyacrylamide gel in reducing conditions (6 mM DTT). Notice that the bulk of host albumin (band of apparent MW 65 kDa, indicated with a big head arrow) and immunoglobulins (bands with apparent MW of about 50 and 25 kDa corresponding to the high and light chains of IgG, respectively, indicated with bold arrows) present in HF were not retained on the Q-Sepharose column and appeared in the flow through fraction (FTf). In addition, an around 8 kDa band (containing likely AgB8 subunits) was slightly stained in HF and undetectable in FTf, but became prominent in the fraction retained by the Q-Sepharose column (QSf). Moreover, the typical AgB pattern with regularly spaced bands is observed in QSf (small head arrows). After the first ultracentrifugation round of QSf, the high (Hdf) and low (Ldf) density fractions were recovered. Ldf contains mainly AgB. B) Analysis of SDS-PAGE of Ldf obtained by two consecutive ultracentrifugation rounds (indicated as 1st UC and 2nd UC) showing that at least two rounds were needed to achieve a good-quality AgB preparation. Simple arrows indicate AgB monomeric and oligomeric forms. (TIF) [file pntd.0005250.s001.tif]
