# Supplementary material for: Characterisation of Antigen B Protein Species Present in the Hydatid Cyst Fluid of Echinococcus canadensis G7 Genotype
Source: PLoS Negl Trop Dis. 2017 Jan 3;11(1):e0005250. doi: 10.1371/journal.pntd.0005250 (PMC5234841; doi:10.1371/journal.pntd.0005250)
Supplement: S1 Table — (PDF) [file pntd.0005250.s002.pdf]

S1 Table. Proteins identified in sQS_f by 2-DGE plus MALDI-TOF/TOF

Spot	Identified protein	Score	N° peptides	% CO
1	AgB subunit 1 [E. canadensis]	121	2	27
	AgB subunit 3 [E. canadensis]	120	2	25
2	AgB subunit 1 [E. canadensis]	164	5	43
	AgB subunit 3 [E. canadensis]	157	2	26
3	AgB subunit 1 [E. canadensis]	109	3	24
4	AgB subunit 1 [E. canadensis]	121	3	26
5	AgB subunit 1 [E. canadensis]	123	2	27
6	AgB subunit 4 [E. canadensis]	330	6	66
	AgB subunit 1 [E. canadensis]	185	4	32
7	AgB subunit 1 [E. canadensis]	159	3	28
8	AgB subunit 4 [E. canadensis]	330	5	66
	AgB subunit 1 [E. canadensis]	164	3	26
9	AgB subunit 4 [E. canadensis]	134	3	29
	AgB subunit 1 [E. canadensis]	128	2	26
10	AgB subunit 4 [E. canadensis]	228	6	62
	AgB subunit 1 [E. canadensis]	185	3	26
11	AgB subunit 1 [E. canadensis]	102	2	29
12	AgB subunit 1 [E. canadensis]	161	3	26
13	AgB subunit 1 [E. canadensis]	126	2	26
14	AgB subunit 1 [E. canadensis]	111	2	29
15	AgB subunit 1 [E. canadensis]	103	2	29
16	AgB subunit 1 [E. canadensis]	129	2	26
	AgB subunit 4 [E. canadensis]	101	5	51
17	AgB subunit 1 [E. canadensis]	105	3	39
18	AgB subunit 4 [E. canadensis]	395	5	60
	AgB subunit 1 [E. canadensis]	172	3	26
19	AgB subunit 4 [E. canadensis]	169	4	45
	AgB subunit 1 [E. canadensis]	103	3	39
20	AgB subunit 1 [E. canadensis]	98	2	26
21	AgB subunit 1 [E. canadensis]	110	2	29
22	AgB subunit 1 [E. canadensis]	118	2	29
23	AgB subunit 1 [E. canadensis]	96	2	29
24	AgB subunit 4 [E. canadensis]	204	5	51
	AgB subunit 1 [E. canadensis]	149	3	33
25	AgB subunit 4 [E. canadensis]	395	4	43
	AgB subunit 1 [E. canadensis]	116	2	26
26	22 kDa antigen 5 [E. canadensis]	173	8	54
27	22 kDa antigen 5 [E. canadensis]	130	7	39
28	22 kDa antigen 5 [E. canadensis]	224	4	32
29	22 kDa antigen 5 [E. canadensis]	511	11	61
30	Apolipoprotein A-I [Sus scrofa]	684	20	63
	22 kDa antigen 5 [E. canadensis]	256	7	40
31	Apolipoprotein A-I [Sus scrofa]	465	17	63

Spot	Identified protein	Score	N° peptides	% CO
	22 kDa antigen 5 [E. canadensis]	131	5	32
32	Immunoglobulin kappa chain [Sus scrofa]	159	2	24
33	Immunoglobulin kappa chain [Sus scrofa]	148	2	26
34	Immunoglobulin lambda chain [Sus scrofa]	192	4	38
35	Immunoglobulin lambda chain [Sus scrofa]	124	5	51
36	Immunoglobulin kappa chain [Sus scrofa]	118	2	24
37	Immunoglobulin lambda chain [Sus scrofa]	270	6	54
38	Immunoglobulin lambda-chain [Sus scrofa]	152	5	51
39	Immunoglobulin kappa chain [Sus scrofa]	118	1	21
40	38 kDa antigen 5 [E. canadensis]	375	11	44
41	38 kDa antigen 5 [E. canadensis]	230	9	38
42	38 kDa antigen 5 [E. canadensis]	202	9	38
43	38 kDa antigen 5 [E. canadensis]	157	6	25
44	38 kDa antigen 5 [E. canadensis]	113	6	23
45	Albumin [Sus scrofa]	563	19	58
