# Supplementary material for: Characterisation of Antigen B Protein Species Present in the Hydatid Cyst Fluid of Echinococcus canadensis G7 Genotype
Source: PLoS Negl Trop Dis. 2017 Jan 3;11(1):e0005250. doi: 10.1371/journal.pntd.0005250 (PMC5234841; doi:10.1371/journal.pntd.0005250)
Supplement: S1 Appendix — Nucleotide sequence for AgB2 (ECANG7_10984) were obtained from E. canadensis genome available at http://parasite.wormbase.org. Putative products of ECANG7_10984 were predicted using the Expasy translate tool (http://web.expasy.org/cgi-bin/translate/dna_aa). (PDF) [file pntd.0005250.s003.pdf]

S1 Appendix

Putative protein products of *E. canadensis AgB2*

Nucleotide sequence for AgB2 (*ECANG7_10984*) were obtained from *E. canadensis* genome available at http://parasite.wormbase.org.

Putative products of *ECANG7_10984* were predicted using the Expasy translate tool (http://web.expasy.org/cgi-bin/translate/dna_aa). Since *ECANG7_10984* contained a substitution in the splicing site (GT-TG instead of GT-AG) translation of protein products was performed considering two possibilities: 1- using the non-canonical TG-GT splice pair, 2- using an upstream AG dinucleotide present in the second exon as the splice acceptor site. Predicted protein products are listed below:

1- Using the non-canonical TG-GT splice pair
5'3' Frame 1
MetRTYILLSLALVAFVAVVQA
KDEPERCKCLITRKLSELRDFFRSDPLGQRLVALGRDLTAICQKLHLKIHEVLKKYVKDLLEEEEEEDDSK**Stop**
5'3' Frame 2
StopGPTSFSLLLSWLSWPSFKLK**Met**SPRDASAS**Stop**RGN**Stop**ANFETSLEVIHWVKDLLLLAGT**Stop**LPSARSCI**Stop**RFTK
C**Stop**RN**Met**SRICWKKKKKR**Met**IQS
5'3' Frame 3
EDLHPSLSCSRGFRGRRSS**Stop**R**Stop**ARE**Met**QVPHNEEIERTSRLL**Stop**K**Stop**STGSKTCCSWQGPDCHLPEAAFEDSRSV
EEICQGFVGRRRRRG**Stop**FKV
3'5' Frame 1
LL**Stop**IILFFFFFQQILDIFLQHFVNLQ**Met**QLLADGSQVPAKSNKSLTQWITSKEVSKFAQFPRYEALASLGLIFSLNDGHESHESK
REKDVGPH
3'5' Frame 2
YFESSSSSSSSNKSLTYFFNTS**Stop**IFKCSFWQ**Met**AVRSLPRATSL**Stop**PSGSLLKKSRSSLNFLV**Met**RHLHLSGSSLA**Stop**T
TATKATRARERR**Met****Stop**VL
3'5' Frame 3
TLNHPLLLLLPTNP**Stop**HISSTLRESSNAASGRWQSGPCQEQQVFDPVDHF**Stop**RSLEVRSISSL**Stop**GTCISRAHL**Stop**LERR
PRKPREQEREGCRSS

2- Using an upstream AG dinucleotide present in the second exon as the splice acceptor site
5'3' Frame 1
MetRTYILLSLALVAFVAVVQA**Stop**ARE**Met**QVPHNEEIERTSRLL**Stop**K**Stop**STGSKTCCSWQGPDCHLPEAAFEDSRSVEEIC
QGFVGRRRRRG**Stop**FKV
5'3' Frame 2
StopGPTSFSLLLSWLSWPSFKHEPERCKCLITRKLSELRDFFRSDPLGQRLVALGRDLTAICQKLHLKIHEVLKKYVKDLLEEEEEE
DDSK**Stop**
5'3' Frame 3
EDLHPSLSCSRGFRGRRSS**Met**SPRDASAS**Stop**RGN**Stop**ANFETSLEVIHWVKDLLLLAGT**Stop**LPSARSCI**Stop**RFTKC**Sto
p**RN**Met**SRICWKKKKKR**Met**IQS
3'5' Frame 1
LL**Stop**IILFFFFFQQILDIFLQHFVNLQ**Met**QLLADGSQPAKSNKSLTQWITSKEVSKFAQFPRYEALASLGL**Met**LERRPRKPREQE
REGCRSS
3'5' Frame 2
YFESSSSSSSSNKSLTYFFNTS**Stop**IFKCSFWQ**Met**AVRSLPRATSL**Stop**PSGSLLKKSRSSLNFLV**Met**RHLHLSGSCLNDGHE
SHESKREKDVGPH
3'5' Frame 3
TLNHPLLLLLPTNP**Stop**HISSTLRESSNAASGRWQSGPCQEQQVFDPVDHF**Stop**RSLEVRSISSL**Stop**GTCISRAHA**Stop**TTAT
KATRARERR**Met****Stop**VL

Open reading frames are highlighted in lightblue
