# Supplementary material for: Characterisation of Antigen B Protein Species Present in the Hydatid Cyst Fluid of Echinococcus canadensis G7 Genotype
Source: PLoS Negl Trop Dis. 2017 Jan 3;11(1):e0005250. doi: 10.1371/journal.pntd.0005250 (PMC5234841; doi:10.1371/journal.pntd.0005250)
Supplement: S2 Appendix — (PDF) [file pntd.0005250.s004.pdf]

S2 Appendix

Analysis of post-translational modifications: phosphorylation and formation of carbonyl groups in AgB subunits

Phosphorylation sites can be predicted in Ser and/or Thr residues in all AgB isoforms (Net Phos 2.0 server, Technical University of Denmark, http://www.cbs.dtu.dk/services/NetPhos/). However, signals corresponding to phosphorylated peptides were not detected by MS. A number of factors usually complicate phosphopeptide identification in complex mixtures using MS/MS approach. In particular, CID of phosphopeptides generates spectra dominated by the neutral loss of the phosphate group and lacking sequence information, thus resulting in reduced-quality MS/MS spectra and lower-confidence in spectral matching for phosphopeptides (Dephoure et al., *Mol Biol Cell* 2013, **24**:535–42).

Formation of carbonyl groups (by oxidative reactions with oxides of nitrogen or metal catalyzed oxidation) was assessed by Western blot taking advantage of their reaction with 2,4-dinitrophenylhydrazine and the detection of the corresponding 2,4-dinitrophenylhydrazone using a commercial specific antibody. Results showed similar levels of carbonylation in AgB8/1-containing spots focused at different pH (see figure below), suggesting that there is no association between carbonylation and acidification of AgB8 subunits. Therefore, further studies are needed to elucidate which molecular modifications may explain the AgB pattern obtained by 2-DGE.

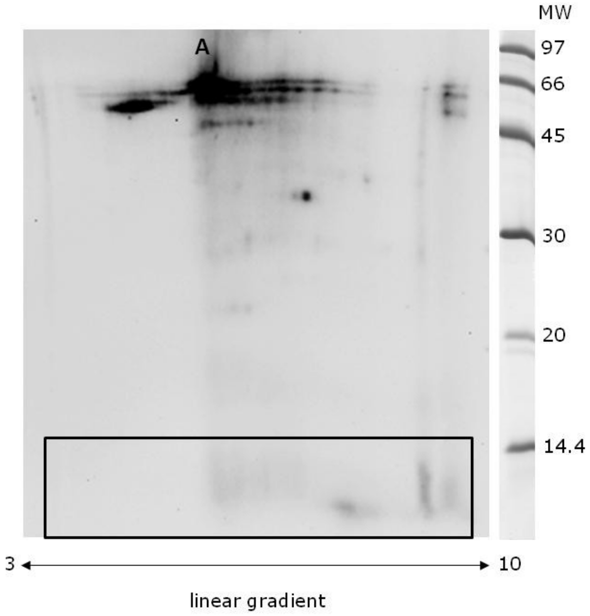

Analysis of AgB carbonylation was performed by 2-DGE using OxyBlot™ Protein Oxidation Detection Kit (S7150, Millipore) with the modifications described previously (Bayot et al., *J Biol Chem* 2010, 285:11445–11457). Briefly, QS_f proteins were electrofocused in a 3-10 pH gradient (as described in Materials and Methods). The carbonyl groups in protein side chains were then derivatized to 2,4-dinitrophenylhydrazone (DNPhydrazone) by reaction with 2,4-dinitrophenylhydrazine. Afterwards, DNP-derivatized proteins were then separated by SDS-PAGE polyacrylamide gel electrophoresis (15%) followed by Western blotting using antibodies specific to the DNP moiety of proteins. Films were scanned using Image Scanner (GE Healthcare). Taking into account the AgB pattern observed by 2-DGE (Figure 1), no positive signals were found to be linked with acidification events.
