# Supplementary material for: Characterisation of Antigen B Protein Species Present in the Hydatid Cyst Fluid of Echinococcus canadensis G7 Genotype
Source: PLoS Negl Trop Dis. 2017 Jan 3;11(1):e0005250. doi: 10.1371/journal.pntd.0005250 (PMC5234841; doi:10.1371/journal.pntd.0005250)
Supplement: S3 Appendix — bQSf proteins species were analysed by DGE followed by MALDI-TOF/TOF while the lipid moiety of bLdf was analysed by HPTLC. (PDF) [file pntd.0005250.s005.pdf]

S3 Appendix

Characterisation of bovine AgB

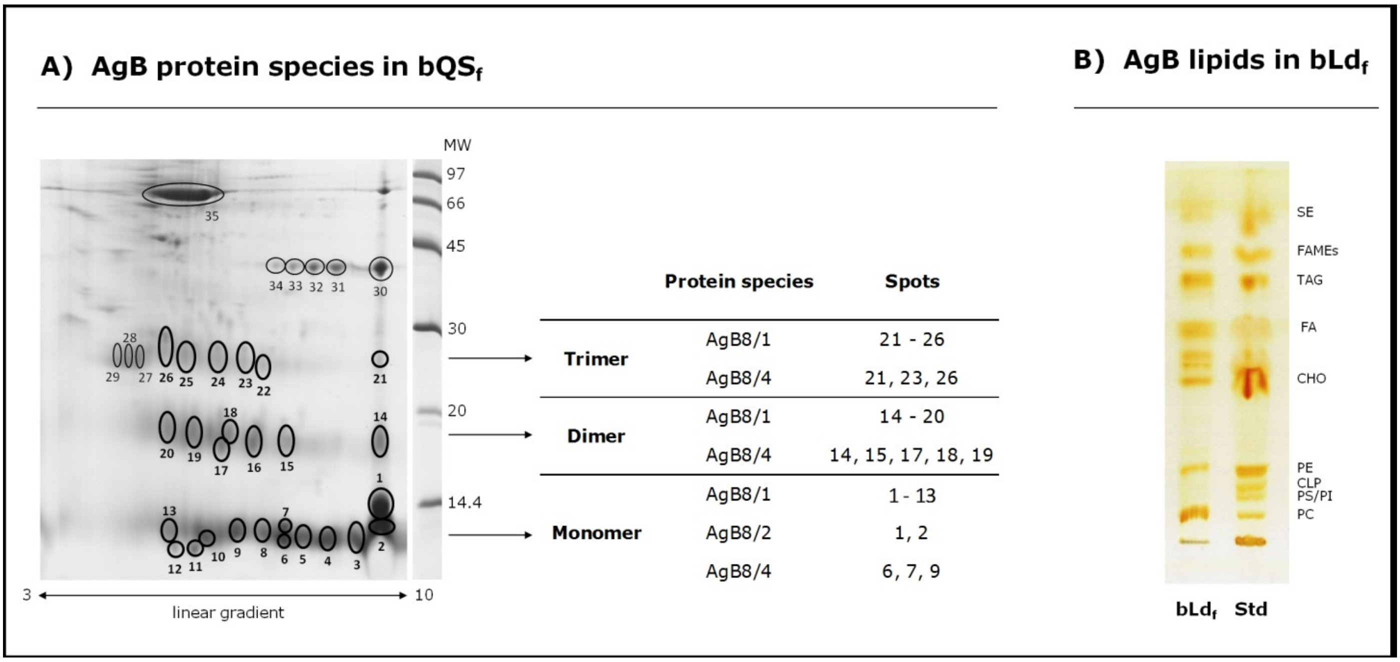

Composition of native AgB: bQSf apolipoproteins identification by 2-DGE plus MALDI-TOF/TOF and bLdf lipid moiety analysis. A) Analysis of bQSf by 2-DGE (figure is representative of analytical triplicates), using a 3-10 lineal gradient of pH in the first dimension, and a 15% acrylamide gel for SDS-PAGE in the second dimension. Gels were stained with colloidal coomassie. The presence of host and parasite components was studied by analysing all spots by MS (MALDI-TOF/TOF). AgB was found in spots regularly spaced at around 8, 16 and 24 kDa (bold circles and arrows). The table illustrates which AgB8 subunits were identified in spots corresponding to the monomeric, dimeric and trimeric forms of AgB. MW: molecular weight (KDa). **B)** Analysis of bLdf by HPTLC (figure is representative of analytical triplicates). Standards and samples (about 10 µg) were applied onto HPTLC plates and resolving using double development solvent system for characterisation of both neutral and polar lipid classes. Lipid bands were visualised using iodine vapour. Std: standard containing polar and neutral lipids; PC: phosphatidylcholine; PS: phosphatidylserine; PI: phosphatidylinositol; CLP: cardiolipin and PE: phsophatidylethanolamine; CHO: cholesterol; FA: free fatty acids; TAG: triacylglycerols; FAMEs: fatty acid methyl esters; SE: sterol esters.

Proteins identified in bQS$_f$ by 2-DGE plus MALDI-TOF/TOF

Spot	Identified protein	Score	N° peptides	% CO
1	AgB subunit 2 [E. granulosus]	154	3	39
	AgB subunit 1 [E. granulosus]	126	3	26
2	AgB subunit 1 [E. granulosus]	125	4	34
	AgB subunit 2 [E. granulosus]	98	2	28
3	AgB subunit 1 [E. granulosus]	125	4	34
4	AgB subunit 1 [E. granulosus]	137	4	34
5	AgB subunit 1 [E. granulosus]	133	5	43
6	AgB subunit 4 [E. granulosus]	235	5	64
	AgB subunit 1 [E. granulosus]	107	3	31
7	AgB subunit 4 [E. granulosus]	155	4	56
	AgB subunit 1 [E. granulosus]	146	4	34
8	AgB subunit 1 [E. granulosus]	154	4	34
9	AgB subunit 4 [E. granulosus]	259	5	64
	AgB subunit 1 [E. granulosus]	102	3	31
10	AgB subunit 1 [E. granulosus]	148	4	34
11	AgB subunit 1 [E. granulosus]	97	3	26
12	AgB subunit 1 [E. granulosus]	112	3	26
13	AgB subunit 1 [E. granulosus]	137	4	34
14	AgB subunit 1 [E. granulosus]	134	4	34
	AgB subunit 4 [E. granulosus]	93	4	43
15	AgB subunit 4 [E. granulosus]	169	5	48
	AgB subunit 1 [E. granulosus]	135	3	26
16	AgB subunit 1 [E. granulosus]	114	3	26
17	AgB subunit 4 [E. granulosus]	207	5	72
	AgB subunit 1 [E. granulosus]	118	3	26
18	AgB subunit 1 [E. granulosus]	175	3	26
	AgB subunit 4 [E. granulosus]	130	5	49
19	AgB subunit 4 [E. granulosus]	90	5	70
	AgB subunit 4 [E. granulosus]	165	4	58
20	AgB subunit 1 [E. granulosus]	99	3	26
21	AgB subunit 1 [E. granulosus]	151	5	39
	AgB subunit 4 [E. granulosus]	107	5	64
22	AgB subunit 1 [E. granulosus]	90	3	26
23	AgB subunit 1 [E. granulosus]	157	4	34
	AgB subunit 4 [E. granulosus]	94	5	48
24	AgB subunit 1 [E. granulosus]	155	4	34
25	AgB subunit 1 [E. granulosus]	139	4	34
26	AgB subunit 4 [E. granulosus]	108	5	48
	AgB subunit 1 [E. granulosus]	93	4	39
27	Apolipoprotein A-I [Bos taurus]	261	15	58
28	22 kDa antigen 5 [E. granulosus]	312	8	57
29	22 kDa antigen 5 [E. granulosus]	268	8	57
30	38 kDa antigen 5 [E. granulosus]	333	13	52
31	38 kDa antigen 5 [E. granulosus]	298	13	52
32	38 kDa antigen 5 [E. granulosus]	276	10	39
33	38 kDa antigen 5 [E. granulosus]	108	9	34
34	38 kDa antigen 5 [E. granulosus]	108	9	34
35	Albumin [Bos taurus]	233	15	47
