# Supplementary material for: Characterisation of Antigen B Protein Species Present in the Hydatid Cyst Fluid of Echinococcus canadensis G7 Genotype
Source: PLoS Negl Trop Dis. 2017 Jan 3;11(1):e0005250. doi: 10.1371/journal.pntd.0005250 (PMC5234841; doi:10.1371/journal.pntd.0005250)
Supplement: S4 Appendix — (PDF) [file pntd.0005250.s006.pdf]

S4 Appendix

Echinococcus proteins identified in sQS_f by LC-MS/MS

Fasta headers or Uniprot accession number	Protein	iBAQ	Score	PSM	Unique peptides	% CO
ECANG7_10738*	AgB subunit 1	27.7	131.7	295	8	55.0
ECANG7_09982#	AgB subunit 4	25.5	55.0	26	4	42.2
Q6VXZ8/Q6VXZ9†	AgB subunit 3	25.2	53.6	37	4	49.2
ECANG7_02369	glycoprotein Antigen 5	23.4	323.3	64	22	58.9
ECANG7_06359	peroxidasin	20.8	304.1	127	19	51.8
ECANG7_10674§	AgB subunit 5	19.8	12.2	11	2	8.0
ECANG7_09876	peroxidasin	19.8	168.3	68	16	29.1
ECANG7_08922	ployU specific endoribonulease	19.3	83.8	43	6	36.2
ECANG7_02915	basement membrane heparan sulfate	19.2	251.7	76	19	14.4
ECANG7_06520	peptidase inhibitor 16	19.1	30.7	13	4	19.8
ECANG7_05702	collagen alpha 1(XV) chain	19.0	97.0	45	5	4.9
ECANG7_04964	proteinase inhibitor I25 cystatin	18.8	133.1	37	9	47.9
ECANG7_05033	14-3-3 protein	18.7	45.7	19	3	13.9
ECANG7_02122	basement membrane heparan sulfate	18.1	323.3	469	136	22.8
ECANG7_00086	estrogen regulated protein EP45	18.0	33.1	23	4	11.6
ECANG7_03384	G protein coupled receptor	18.0	78.0	26	5	14.7
ECANG7_10405	immunogenic protein	17.9	11.9	8	2	13.5
ECANG7_00937	beta hexosaminidase subunit alpha	17.8	29.2	18	3	7.0
ECANG7_03451	lysosomal alpha mannosidase	17.6	295.6	64	18	22.2
ECANG7_05502	lysosomal Pro X carboxypeptidase	17.5	21.2	21	3	7.4
ECANG7_05731	14-3-3 protein homolog 2	17.5	44.6	20	3	22.5
ECANG7_06318	Abnormal EMBroygenesis family member emb 9	17.3	105.2	36	8	6.0
ECANG7_07681	expressed conserved protein	17.3	17.0	8	2	5.8
ECANG7_01963	egf domain protein	17.2	323.3	121	31	15.7
ECANG7_11187	alfa 2 macroglobulin	17.2	76.2	17	9	11.8
ECANG7_06422	collagen alpha 1(XV) chain	17.1	38.7	16	4	3.5
ECANG7_07672	phosphoenolpyruvate carboxykinase	16.8	67.4	33	5	7.1
ECANG7_03962	laminin	16.5	323.3	165	46	17.9
ECANG7_08217	notch	16.1	27.8	12	3	2.9
ECANG7_01427	laminin subunit beta	15.9	233.6	57	13	10.8
ECANG7_04980	putative alpha 2 macroglobulin	15.8	24.1	11	4	3.0
ECANG7_08998	Amine oxidase	17.3	32.1	29	2	2.2
ECANG7_01260	lipid transport protein N terminal	15.1	101.6	37	10	5.8
ECANG7_08193	protocadherin 1	14.5	20.0	8	2	1.8
ECANG7_04889	collagen alpha (IV) chain	13.3	18.1	4	7	4.9
ECANG7_04822	Fras1 related extracellular matrix protein	13.2	30.2	23	6	4.1

The following symbols indicate that these genes encode the protein products annotated as: * Q86BY8 and Q3YFQ5, # Q6Q0G7 and Q6Q0G2 and § Q1EQ64
† The gen that encodes these protein products could not be identified in *E. canadensis* genome (http://parasite.wormbase.org as echinococcus_canadensis.PRJEB8992.WBPS5.protein)

Host proteins identified in sQS$_f$ by LC-MS/MS

Fasta headers /Uniprot accession number	iBAQ	Score	PSM	Unique peptides	% CO
>tr|F1RUN2|F1RUN2_PIG Serum albumin	26.5	323.3	431	38	70.8
>tr|K7ZJP7|K7ZJP7_PIG IgM heavy chain constant region	25.4	245.6	140	17	66.5
>tr|I3LAQ0|I3LAQ0_PIG Uncharacterized protein	24.2	129.9	52	6	42.0
>sp|P01846|LAC_PIG Ig lambda chain C region	24.0	72.2	27	3	84.8
>sp|P18648|APOA1_PIG Apolipoprotein A-I	23.2	116.7	40	9	50.8
>tr|K7ZRK0|K7ZRK0_PIG IgA heavy chian constant region	23.1	143.6	64	11	64.0
>tr|F1RUQ0|F1RUQ0_PIG Uncharacterized protein	22.7	45.8	20	4	33.5
>tr|I3VKE6|I3VKE6_PIG Ceruloplasmin	22.3	122.9	127	22	37.6
>sp|Q29549|CLUS_PIG Clusterin	22.2	323.3	148	28	43.2
>sp|P02067|HBB_PIG Hemoglobin subunit beta	21.9	89.2	29	6	64.6
>sp|P00761|TRYP_PIG Trypsin	21.2	48.9	44	3	22.5
>sp|P01025|CO3_PIG Complement C3	21.1	323.3	77	15	55.9
>tr|F1RKY2|F1RKY2_PIG Uncharacterized protein	21.1	84.2	38	9	19.5
>tr|F1RX35|F1RX35_PIG Uncharacterized protein	21.0	130.8	52	12	36.4
>tr|F1SS24|F1SS24_PIG Uncharacterized protein	21.0	323.3	108	16	34.7
>tr|Q68RU1|Q68RU1_PIG Ovarian and testicular apolipoprotein N	20.6	35.8	17	4	16.2
>tr|A5A8W8|A5A8W8_PIG Complement component 4A	20.5	323.3	179	38	37.9
>tr|I3L638|I3L638_PIG Vitronectin	20.5	78.8	32	8	25.1
>tr|F1SFI4|F1SFI4_PIG Uncharacterized protein	20.4	61.3	23	6	16.1
>tr|L8B0S2|L8B0S2_PIG IgG heavy chain	20.3	75.3	19	4	27.8
>tr|F1RX37|F1RX37_PIG Fibrinogen beta chain	20.1	141.5	40	10	37.6
>tr|F1S0J2|F1S0J2_PIG Uncharacterized protein	20.1	74.8	32	8	16.6
>tr|I3LF89|I3LF89_PIG Uncharacterized protein	20.1	94.2	41	10	30.1
>sp|P19133|FRIL_PIG Ferritin light chain	20.1	16.7	8	2	39.7
>tr|I3LN42|I3LN42_PIG Uncharacterized protein<br>>tr|Q66RQ0|Q66RQ0_PIG Vitamin D-binding protein	20.0	157.2	42	9	35.1
>tr|F1RX36|F1RX36_PIG Fibrinogen alpha chain	19.7	63.4	15	4	26.2
>sp|O02668|ITIH2_PIG Inter-alpha-trypsin inh. heavy chain H2	19.7	95.9	60	11	17.1
>tr|F1SCD0|F1SCD0_PIG Uncharacterized protein	19.7	73.8	27	7	24.4
>tr|B3STX9|B3STX9_PIG Prothrombin	19.4	113.3	31	7	20.9
>tr|L8B0W0|L8B0W0_PIG IgG heavy chain	19.4	61.6	12	2	32.5
>tr|F1SQ09|F1SQ09_PIG Uncharacterized protein	19.4	65.4	15	4	25.1
>tr|V5L1C3|V5L1C3_PIG POSTN	19.3	128.7	53	11	22.6
>tr|I3LRJ4|I3LRJ4_PIG Vitamin K-dependent protein C	19.2	68.5	24	6	16.0
>tr|B1PSB6|B1PSB6_PIG Adiponectin	19.2	38.8	13	3	22.7
>sp|P27917|APOC3_PIG Apolipoprotein C-III	19.1	32.3	65	2	28.1
>tr|F1RN76|F1RN76_PIG Uncharacterized protein	19.1	47.5	15	4	11.5
>tr|F1RM45|F1RM45_PIG Apolipoprotein E	19.0	74.6	20	5	19.0
>tr|I3LQ17|I3LQ17_PIG Uncharacterized protein	18.9	162.5	58	14	11.7
>tr|I3LTB8|I3LTB8_PIG Uncharacterized protein	18.9	11.5	6	2	58.8
>sp|P16293|FA9_PIG Coagulation factor IX	18.9	40.1	15	4	15.8
>tr|F1S1A9|F1S1A9_PIG Uncharacterized protein	18.8	27.1	7	2	22.0
>sp|P04366|AMBP_PIG Protein AMBP	18.6	18.3	8	2	8.9
>sp|Q2LE37|APOM_PIG Apolipoprotein M	18.4	25.1	8	2	12.5
>sp|Q6QAQ1|ACTB_PIG Actin, cytoplasmic 1	18.2	130.9	24	3	36.9
>tr|F1SQX9|F1SQX9_PIG Apolipoprotein D	18.1	17.0	6	2	10.0
>tr|F1RFY1|F1RFY1_PIG Profilin	18.0	25.4	9	2	21.8
>tr|A4US67|A4US67_PIG Paraoxonase	17.9	50.6	10	2	13.6

Fasta headers / Uniprot accession number	iBAQ	Score	PSM	Unique peptides	% CO
>tr|K7ZPU8|K7ZPU8_PIG IgG heavy chian constant region	17.8	17.5	8	2	34.9
>tr|Q6VPV1|Q6VPV1_PIG Complement component C5	17.8	129.7	48	11	10.9
>sp|Q29052|ITIH1_PIG Inter-alpha-trypsin inh.heavy chain H1	17.8	146.7	12	3	16.2
> tr|K7GNI9|K7GNI9_PIG Phosphoglycerate kinase	17.5	13.1	7	2	10.8
> tr|F2Z558|F2Z558_PIG Uncharacterized protein	17.5	89.0	30	3	19.7
>tr|F1SFI5|F1SFI5_PIG Uncharacterized protein	17.4	63.5	18	5	9.7
> tr|A0SEG9|A0SEG9_PIG Complement component C9	16.9	13.5	6	2	3.1
>tr|F1RI39|F1RI39_PIG Uncharacterized protein	16.7	64.5	42	6	7.3
> tr|Q7M364|Q7M364_PIG Antithrombin III	16.5	31.1	10	3	7.6
>tr|F1SH96|F1SH96_PIG Uncharacterized protein	16.2	21.9	8	2	18.6
> tr|I3LK59|I3LK59_PIG Uncharacterized protein	16.1	18.9	6	2	5.3
>tr|F1S682|F1S682_PIG Sulfhydryl oxidase	15.8	28.1	14	4	5.7
> tr|F1SB81|F1SB81_PIG Plasminogen	15.5	20.5	10	3	3.4
>tr|F1SHL9|F1SHL9_PIG Pyruvate kinase	15.5	32.0	9	2	5.2
>sp|Q69DK8|C1S_PIG Complement C1s subcomponent	15.5	25.8	11	2	3.3
>tr|F1RK02|F1RK02_PIG Uncharacterized protein	15.4	172.1	9	2	35.4
>tr|I3LAQ4|I3LAQ4_PIG Uncharacterized protein	15.3	15.0	8	2	5.8
>tr|F1SCV9|F1SCV9_PIG Uncharacterized protein	14.8	32.9	10	3	4.1
>tr|Q29021|Q29021_PIG Apolipoprotein B	14.6	77.0	26	6	3.5

Echinococcus proteins identified in bQS$_f$ by LC-MS/MS

Fasta headers / Uniprot accession numbers	iBAQ	Score	PSM	Unique peptides	% CO
>EgrG_000381200.1.pep|*E. granulosus* Antigen B subunit 1*; Q5EKQ4_ECHGR; Q3YFP9_9CEST	29.1	162.3	363	10	72.6
>EgrG_000381700.1.pep|*E. granulosus* Antigen B subunit 3; Q95NW6_ECHGR[#]	25.2	49.6	32	4	50.2
>EgrG_000184900.1..pep|glycoprotein Antigen 5	24.4	323.3	540	26	66.3
Q3YFP3_9CEST| *E. ortleppi* Antigen B subunit 3	22.5	36.1	14	4	24.6
>EgrG_000381100.1.pep|*E. granulosus* Antigen B subunit 2[§]; Q5EKP1_ECHGR	20.9	75.8	16	8	60.6
>EgrG_000549200.1..pep|hydrophobic ligand binding protein[‡‡‡]	19.5	18.4	9	2	20.5
>EgrG_000550400.1..pep|expressed protein	19.0	14.0	13	2	32.2
>EgrG_000381400.1.pep|*E. granulosus* Antigen B subunit 4[†]	19.0	77.0	6	9	76.8
>EgrG_000682900.1..pep|niemann Pick C2 protein	18.8	41.7	17	2	15.8
>EgrG_000381700.1.pep|E. granulosus Antigen B subunit 5[‡‡]	18.7	13.3	5	2	17.2
Q6UZE2[‡]; Q6UZD8 *E. granulosus* AgB subunit 4	18.4	58.6	7	9	78.6
>EgrG_000354700.1..pep|tetraspanin	18.4	24.3	15	2	9.6
>EgrG_000733100.1..pep|prosaposin a preproprotein	18.2	39.7	39	4	4.7
>EgrG_000849600.1..pep|proteinase inhibitor I25 cystatin	18.2	174.2	85	9	45.5
>EgrG_000791700.1..pep|thioredoxin peroxidase	18.1	28.6	45	4	19.5
>EgrG_000575900.1..pep|basement membrane heparan sulfate	17.9	323.3	1066	116	20.8
>EgrG_000355700.1..pep|tetraspanin	17.8	32.8	13	3	12.7
>EgrG_000244000.1..pep|annexin	17.7	61.0	24	4	15.8
>EgrG_000041200.1..pep|annexin	17.5	27.3	19	3	9.9
>EgrG_000530400.1..pep|amiloride sensitive amine oxidase	17.5	32.1	29	4	5.1
>EgrG_001132700.1..pep|polyu specific endoribonuclease	17.5	83.8	43	4	21.2
>EgrG_000523100.1..pep|expressed conserved protein	17.4	57.2	19	2	4.4
>EgrG_000143500.1..pep|lysosomal alpha glucosidase	16.9	54.4	26	5	7.7
>EgrG_001028500.1..pep|citrate synthase	16.8	28.6	21	3	8.0
>EgrG_000701800.1..pep|basement membrane heparan sulfate	16.7	301.9	14	2	21.8
>EgrG_000729300.1..pep|collagen alpha 1(XV) chain	16.5	97.0	47	7	5.9
>EgrG_000364000.1..pep|14-3-3 protein beta:alpha	16.4	44.6	20	2	13.6
>EgrG_000684200.1..pep|lipid transport protein N terminal	16.3	323.3	162	28	11.2
>EgrG_000733600.1..pep|peroxidasin	16.0	304.1	127	9	9.5
>EgrG_001192500.1..pep|14-3-3 protein	16.0	45.7	24	2	7.1
>EgrG_000456150.1..pep|lysosomal Pro X carboxypeptidase	15.7	21.2	21	2	5.7
>EgrG_001200300.1..pep|desert hedgehog protein	15.5	51.0	18	3	10.1
>EgrG_000903100.1..pep|calsyntenin 1	15.4	39.1	13	3	2.4
>EgrG_001032250.1..pep|aminotransferase class III; >EgrG_001032200.1..pep|ornithine aminotransferase	15.3	19.9	8	2	6.4
>EgrG_000704400.1..pep|lysosomal alpha mannosidase	15.2	39.9	21	4	17.0
>EgrG_000789900.1..pep|beta mannosidase	15.2	43.6	19	4	5.4
>EgrG_000824000.1..pep|estrogen regulated protein EP45	15.2	33.1	23	2	7.4
>EgrG_000292700.1..pep|phosphoenolpyruvate carboxykinase	15.0	67.4	44	4	6.5
>EgrG_001169400.1..pep|iron:zinc purple acid phosphatase protein	14.6	78.0	26	2	4.7
>EgrG_000524200.1..pep|collagen type XI alpha 2	14.4	34.3	13	3	2.6
>EgrG_000343000.1..pep|neurogenic locus notch protein	14.4	73.4	35	5	2.1
>EgrG_000255800.1..pep|egf domain protein	13.6	32.3	96	2	2.4
>EgrG_001132400.1..pep|laminin	13.5	23.5	8	2	3.0

The following symbols indicate that the protein product has also been annotated as:
* Q9UA06, U6JQF4 and Q5S577
[#] Q5EKQ8, Q5EKR1, Q5EKR3 and Q95W92
[§] Q27275, Q5EKN4, C1KBK4, Q6Q0H3 and Q6Q0I3
[†] Q6UZE3, D1MH02
[‡] Q6GYC5
[‡‡] D1MH21, U6JQF8 and Q1EQ65
[‡‡‡] A0A068WMS7

Host proteins identified in bQS_f by LC-MS/MS

<table>
<tr><th>Fasta headers / Uniprot accession numbers</th><th>iBAQ</th><th>Score</th><th>PSM</th><th>Unique peptides</th><th>% CO</th></tr>
<tr><td>>tr|F1N6C0|F1N6C0_BOVIN Uncharacterized protein
>sp|P62157|CALM_BOVIN Calmodulin</td><td>21.8</td><td>104.1</td><td>50</td><td>7</td><td>49.1</td></tr>
<tr><td>>sp|P02769|ALBU_BOVIN Serum albumin</td><td>20.8</td><td>290.9</td><td>46</td><td>4</td><td>63.9</td></tr>
<tr><td>>tr|G5E5T5|G5E5T5_BOVIN Uncharacterized protein</td><td>20.3</td><td>102.2</td><td>57</td><td>9</td><td>23.4</td></tr>
<tr><td>>tr|A6QNZ7|A6QNZ7_BOVIN Keratin 10</td><td>20.0</td><td>243.3</td><td>105</td><td>13</td><td>30.1</td></tr>
<tr><td>>sp|P15497|APOA1_BOVIN Apolipoprotein A-I</td><td>19.9</td><td>51.4</td><td>33</td><td>5</td><td>34.6</td></tr>
<tr><td>>tr|D4QBB4|D4QBB4_BOVIN Hemoglobin beta</td><td>19.5</td><td>94.6</td><td>37</td><td>6</td><td>52.0</td></tr>
<tr><td>>tr|F1MS32|F1MS32_BOVIN Apolipoprotein D</td><td>18.9</td><td>29.9</td><td>15</td><td>3</td><td>20.6</td></tr>
<tr><td>>sp|P62261|1433E_BOVIN 14-3-3 protein epsilon</td><td>18.5</td><td>53.8</td><td>26</td><td>4</td><td>25.1</td></tr>
<tr><td>>tr|Q1RMN8|Q1RMN8_BOVIN Immunoglobulin light chain</td><td>18.3</td><td>73.5</td><td>26</td><td>8</td><td>25.0</td></tr>
<tr><td>>tr|F1MU12|F1MU12_BOVIN Keratin, type II cytoskeletal 8</td><td>17.6</td><td>82.5</td><td>33</td><td>7</td><td>19.7</td></tr>
<tr><td>>sp|Q9TTE1|SPA31_BOVIN Serpin A3-1</td><td>17.5</td><td>78.0</td><td>20</td><td>3</td><td>12.5</td></tr>
<tr><td>>tr|R9QSM8|R9QSM8_BOVIN Alpha-2-macroglobulin</td><td>17.2</td><td>119.0</td><td>53</td><td>11</td><td>13.0</td></tr>
<tr><td>>sp|P63103|1433Z_BOVIN 14-3-3 protein zeta/delta</td><td>17.1</td><td>89.0</td><td>30</td><td>3</td><td>24.0</td></tr>
<tr><td>>sp|Q2UVX4|CO3_BOVIN Complement C3</td><td>16.6</td><td>170.5</td><td>48</td><td>10</td><td>11.2</td></tr>
<tr><td>>sp|P63258|ACTG_BOVIN Actin, cytoplasmic 2</td><td>16.5</td><td>130.9</td><td>24</td><td>3</td><td>30.3</td></tr>
<tr><td>>tr|A6QR15|A6QR15_BOVIN LOC535277 protein</td><td>16.4</td><td>27.8</td><td>11</td><td>2</td><td>14.5</td></tr>
<tr><td>>tr|I7CT57|I7CT57_BOVIN Vitamin D binding protein</td><td>16.3</td><td>30.5</td><td>14</td><td>3</td><td>12.8</td></tr>
<tr><td>>sp|P00978|AMBP_BOVIN Protein AMBP</td><td>16.3</td><td>47.3</td><td>10</td><td>2</td><td>9.2</td></tr>
<tr><td>>sp|Q5KR48-2|TPM2_BOVIN Tropomyosin beta chain</td><td>16.2</td><td>18.0</td><td>8</td><td>2</td><td>9.7</td></tr>
<tr><td>>sp|P48644|AL1A1_BOVIN Retinal dehydrogenase 1</td><td>16.0</td><td>26.8</td><td>12</td><td>2</td><td>5.6</td></tr>
<tr><td>>sp|P00735|THRB_BOVIN Prothrombin</td><td>16.0</td><td>38.6</td><td>10</td><td>2</td><td>5.3</td></tr>
<tr><td>>tr|A5D7Q2|A5D7Q2_BOVIN Putative uncharacterized protein</td><td>15.7</td><td>40.5</td><td>11</td><td>2</td><td>7.9</td></tr>
<tr><td>>tr|B0JYQ0|B0JYQ0_BOVIN ALB protein</td><td>14.8</td><td>17.4</td><td>13</td><td>2</td><td>57.6</td></tr>
<tr><td>>tr|A4ZZF8|A4ZZF8_BOVIN Alpha-actinin 1</td><td>14.8</td><td>64.5</td><td>42</td><td>4</td><td>4.7</td></tr>
</table>
