# Supplementary material for: Characterisation of Antigen B Protein Species Present in the Hydatid Cyst Fluid of Echinococcus canadensis G7 Genotype
Source: PLoS Negl Trop Dis. 2017 Jan 3;11(1):e0005250. doi: 10.1371/journal.pntd.0005250 (PMC5234841; doi:10.1371/journal.pntd.0005250)
Supplement: S5 Appendix — (PDF) [file pntd.0005250.s007.pdf]

S5 Appendix

A) Alignments of HLBPs from *Echinococcus* A0A068WMS7_EGHR and W6UNU2_ECHGR with their orthologous in *Taenia solium.* **B)** Alignment of *Echinococcus* HLBPs A0A068WMS7_EGHR with AgB subunits.

A)

```
TR|A0A068WMS7|A0A068WMS7_ECHGR MRAGIVALLTILAVVAVSAEEKNPIRDIRARYK----AKLEKWE-------KFFEEDPLG 49
TR|W6UNU2|W6UNU2_ECHGR         MRAGIVALLTILAVVAVSAE---------ARYK----TKLEKWE-------KFFEEDPLG 40
TR|G3FJ94|G3FJ94_TAESO         MRVAVIALLALLAVVAVSA-EKNPVRVIRTKYK----EKVEKWE-------KFFDEDPLG 48
TR|G3FJ95|G3FJ95_TAESO         MRVAVIALLALLAVVAVSA-EKNPVRVIRTKYK----EKVEKWE-------KFFDEDPLG 48
TR|A0A068Y7J9|A0A068Y7J9_ECHMU MRTVIVALLTVLAVVAVSAEEVSPTTQSSTLYFPIRKSKPRPWQKSPIQVGKVLRGGPSR 60
                               **. ::***::********          : *      * . *:       *.:   *

TR|A0A068WMS7|A0A068WMS7_ECHGR QKISDHYAELKELIKEVRQRIRKQLAKYIKQLKSE--- 84
TR|W6UNU2|W6UNU2_ECHGR         QKISDHYAELKELIKEVRQRIRKQLAKYVKQLKSE--- 75
TR|G3FJ94|G3FJ94_TAESO         QKIADHYAKLRELVKEVKLRIRKAIAKCVKKLEDGGDD 86
TR|G3FJ95|G3FJ95_TAESO         QKIADHYAKLRELVKEVKLRIRKAIAKYVKELKSEN-- 84
TR|A0A068Y7J9|A0A068Y7J9_ECHMU PKISDHYAKLKELIKDVRQRICKHLAKYVKQLGSE--- 95
                                **:****:*:**:*:*: ** * :** :*:* .
```

B)

```
TR|A0A068WMS7|A0A068WMS7_ECHGR MRAGIVALLTILAVVAVSAEEKNP--IRDIRARYKAKLEKWEKFFEEDPLGQKISDHYAE 58
TR|Q24790|Q24790_ECHGR         ----MLLALALVSFVVVT---QADDGLTSTSRSVMKMIGERKYFFERDPLGQKVVDLLKE 53
TR|Q27275|Q27275_ECHGR         MRTYILLSLALVAFVAVVQAKDEPK--AHMGQVVKKRWGELRDFFRNDPLGQRLVALGND 58
TR|Q95NW6|Q95NW6_ECHGR         -------------FVVVAHADDDDEVTKTKKGVMKAISEIKHFFQSDPLGKKLVEVMKD 47
TR|Q6UZE1|Q6UZE1_ECHGR         ---------ALVAFVAVVQAKAEPE--RCK-CLIMRKLGEIRDVFRSDPLGQKLVALGRD 48
TR|D1MH21|D1MH21_ECHGR         ---------------------EDDIDSKAKKGVMKSVAELKEFFASDPMGQKLASICKE 38
                                                                      : . .*  **:*:::     :

TR|A0A068WMS7|A0A068WMS7_ECHGR LKELIKEVRQRIRKQLAKYIKQLKSE------- 84
TR|Q24790|Q24790_ECHGR         LEEVFQLLRKKLRTALKSHLRELVAEGK----- 81
TR|Q27275|Q27275_ECHGR         LTAICQKLQLKIREVLKKYVKNLVEEKDDDSK- 90
TR|Q95NW6|Q95NW6_ECHGR         VASVCEMVRKKARMALKEYVRKLVKEDE----- 75
TR|Q6UZE1|Q6UZE1_ECHGR         LTAICQKLQLKVHEVLKKYVKDLLEEEDEDDLK 81
TR|D1MH21|D1MH21_ECHGR         LKDFFLLARTKARSALRDYVKRLMDEGE----- 66
                               :  .    : : :  * .::: *  *
```
